# Argon I Lines Produced in a Hollow Cathode Source, *332 nm* to *5865 nm*

**DOI:** 10.6028/jres.10714

**Published:** 2002-04-01

**Authors:** W. Whaling, W. H. C. Anderson, M. T. Carle, J. W. Brault, H. A. Zarem

**Affiliations:** California Institute of Technology, Pasadena CA; National Solar Observatory,^1^ Tucson, AZ

**Keywords:** argon, atomic spectra, Fourier transform spectroscopy, wavelengths, pressure shifts

## Abstract

We report precision measurements by Fourier transform spectroscopy of the vacuum wavenumber, line width, and relative signal strength of 928 lines in the Ar I spectrum. Wavelength in air and classification of the transition are supplied for each line. A comparison of our results with other precision measurements illustrates the sensitivity of Ar I wavelengths to conditions in the light source.

## Foreword

Spectra of the noble gases are frequently observed in laboratory discharges, industrial plasmas, lighting discharges, sources for analytical spectrochemistry, and astrophysical sources. In response to the wide interest in these elements the NIST Atomic Spectroscopy Data Center is currently compiling the spectra and energy levels for all ionization stages of Ne, Ar, Kr, and Xe. A critical review of the literature revealed that no comprehensive description of the infrared spectrum of neutral argon has ever been published.

In 1995 observations of the spectrum of singly ionized argon covering the range 222 nm to 5865 nm were published by Whaling, Anderson, Carle, Brault, and Zarem [J. Quant. Spectros. Radiat. Transfer **53**, 1 (1995)]. Their measurements, made with the high-resolution Fourier Transform Spectrometer at the National Solar Observatory (Kitt Peak), have been widely used as a source of wavelength standards. Measurements of neutral argon from the same spectra have been circulated as an unpublished line list. This list has been used in a number of laboratories to identify lines of argon in spectral sources and as a source of calibration wavelengths.

This article presents the comprehensive list of neutral argon observations from the Kitt Peak spectra over a wide wavelength region from the near ultraviolet to near infrared and describes the experimental conditions under which the measurements were made. It was prepared by Ward Whaling for publication in the NIST Journal of Research with the encouragement and assistance of the Atomic Spectroscopy Data Center in order to make these data available to the broad community of users and to adequately document the work so that its results can be integrated with other available data in our argon compilation.

Craig J. Sansonetti

NIST Atomic Spectroscopy Data Center

## 1. Introduction

Argon is frequently used to sustain the discharge in a hollow cathode spectral source, and the source typically excites the first, second, and third spectra of argon as well as the spectrum of the cathode material. Consequently, a complete picture of the argon spectrum is useful in avoiding, or correcting for, interference between argon lines and lines from the cathode material. In this paper we provide such a line list for Ar I; similar line lists for Ar II [1] and Ar III [2] have been published earlier.

## 2. Experimental Method

Argon lines were obtained by comparing many high-resolution spectra of a hollow cathode discharge in argon, with *different* cathode materials for the different spectra. By extracting lines common to several spectra, regardless of the cathode material, we have generated a list of lines arising from the argon that supports the discharge, or from a common contaminant as discussed below.

All spectra were recorded on the 1 m vacuum Fourier transform spectrometer [3] at the National Solar Observatory (Kitt Peak). Hollow cathode spectra from the Kitt Peak archive with cathodes of Co, Cu, Fe, Mo, Ti, V, and Y were measured for this work; the Kitt Peak National Observatory archive designation of each spectrum is given in Ref. [1]. The cathode cavity was 25 mm long by 8 mm diameter; further details of the water-cooled source will be found in Ref. [1]. The source pressure varied from one spectrum to another between 270 Pa and 530 Pa (2 Torr and 4 Torr) of high purity argon, with discharge currents between 150 mA and 600 mA.

All spectra were measured with the DECOMP [4] line-finding program developed by Brault. This program fits a Voigt profile to the spectral feature and records the line-center, peak amplitude, full width at half maximum intensity (FWHM), and other parameters of the Voigt profile.

## 3. Results

In the first column of Table 1 we list the line-center vacuum wavenumber (in cm^−1^), followed by the experimental uncertainty in column 2. The ratio of the peak amplitude *S* to the root mean square background noise *N* appears in column 3 as the log_10_(*S*/*N*) for each line. Note that the peak amplitude has not been corrected for the spectrometer response. Over the range of a given spectrum, the spectrometer response may vary by an order of magnitude, and the comparison of the signal-to-noise ratio of two lines is a reliable indication of the relative strength of the lines only if they are close in wavelength. The full width of the observed line (in 10^−3^ cm^−1^) at half maximum height appears in the 4th column. The absolute value of the line width depends on source and instrumental conditions, but the increased relative width of lines from levels of high excitation and high angular momentum is an aid in identification.

The uncertainty Δ*σ* in the line-center wavenumber appears in the second column. This experimental uncertainty (one standard deviation level) comes from two sources: (1) the uncertainty Δ*σ*_L_ in locating the center of the line in the presence of noise, and (2) Δ*σ*_S_ from the uncertainty in the scale of the FTS. We combine these to find the overall uncertainty in the wave number: Δ*σ* = [(Δ*σ*_L_)^2^ + (Δ*σ*_S_)^2^)]^1/2^.

1. We evaluate the uncertainty in locating the line center from Δ*σ*_L_ = 0.5(*W*)/(*S*/*N*), where *W* (the FWHM) and *S*/*N* (the signal-to-noise ratio) appear in Table 1, and the factor 0.5 is an approximation to the more precise factor given by Davis, Abrams, and Brault [5]. For very strong lines (*S*/*N* > 10^4^) this expression may underestimate Δ*σ*_L_ because it neglects self-absorption that may, if asymmetric, shift the line center, and it neglects the local increase in the noise level under a strong line in the FTS spectrum [6]. We therefore set a lower limit on Δ*σ*_L_ of 0.0003 cm^−1^. The argument supporting this limiting value is presented in Ref. [1].
2. Wavenumbers measured with the FTS must be multiplied by a scale factor *s* to correct for any angular deviation between the path followed by light from the source as it passes through the instrument and the path followed by the laser beam that measures the displacement of the FTS mirror. The scale factor *s* is a constant (~1) for a given spectrum and is determined empirically by measuring with the FTS standard lines of accurately known wavenumber; *s* = (standard wavenumber)/(measured wavenumber). Any uncertainty Δ*s* in the determination of *s* contributes a proportional uncertainty in the corrected wavenumber *σ* given by Δ*σ*_S_ = *σ*(Δ*s*/*s*). The factor in parentheses was typically (3 to 7) × 10^−8^ for the different spectra that we measured. We adopt as the uncertainty Δ*s* for a particular spectrum the standard deviation in the mean value of *s* derived from measurements of 28 Ar II standard lines in that spectrum.

As standards we used as many as possible of the 28 Ar II lines recommended by Learner and Thorne [7]. These well isolated lines of good strength from transitions between low excitation levels should be minimally sensitive to pressure in the source. Our wavenumbers for these Ar II standard lines were taken from Ref. [1] and derive ultimately from the CO molecular lines used to calibrate the Ar II spectra [8]. Our Ar II wavenumbers are slightly higher than the values reported by Norlén [9]. For the 28 Ar II standard lines, the mean value of the ratio *σ* (based on CO)/(Norlén) is [1 + 67(8) × 10^−9^].

The list of argon wavenumbers collected by comparing different spectra was then compared with line lists in the literature for Ar I, Ar II, and Ar III—notably those of Minnhagen [10], Striganov and Sventitskii [11], and Kurucz and Peytremann [12]—to identify the ion and classify the transition. Additional Ar I lines with wavelengths longer than those in existing compilations were identified by calculating all electric dipole transition energies allowed by parity and *J*-value selection rules, using Ar I level energies from Moore [13] as expanded and refined by level energies from Minnhagen [10] and Norlén [9]. In Table 1, we identify the transition upper level in columns 5–7 by its energy, *J*-value, and orbital, and the lower level in columns 8–10. When individual *J* levels could not be resolved, the [K] value (the result of coupling the total angular momentum *J*_1_ of the 3*p*^5^ core with the orbital angular momentum *l* of the valence electron) is given. The last column gives the wavelength in air as determined from the vacuum wavenumber by a formula from Edlén [14].

Ar II and Ar III lines were identified and classified in the same way. When all Ar I, II, and III lines had been removed from the list of common lines, we searched for possible contaminants that might be common to all our spectra by searching for the strongest lines in the spectra of H, He, C, N, O, Ne, Na, and Fe. A few H, C, and O lines were found, readily identified as coming from a low-mass contaminant by their broad Doppler widths. On the Cu cathode spectrum between 2000 cm^−1^ and 3000 cm^−1^, molecular lines from ArH^+^ were abundant and strong (*S*/*N* up to 10^2^).

With all known Ar and contaminant lines removed, the 114 common lines left over are presumably from Ar. By comparing the relative intensity of these unidentified lines in spectra recorded at different power levels, we conclude that most come from an Ar ion. Four unidentified lines that appear to come from Ar I are included in Table 1 with no designation.

## 4. Comparison With Other Line Lists

Our Ar I wavelengths agree with those of Minnhagen [10] within the modest precision he assigns: “better than 10 mÅ for lines that are not too weak.” Our agreement with Norlén’s [9] wavenumbers is generally within (1 or 2) × 10^−3^ cm^−1^, as might be expected from the close agreement of his calculated wavenumbers for Ar II with the values we used to calibrate the FTS scale. Norlén’s calculated wavenumbers are essentially averages over many alternate (Ritz-equivalent) paths that reduce the uncertainty of his values well below that which we are able to achieve in a single measurement, especially for weak lines. Hence Norlén’s values, multiplied by [1 + 67(8) × 10^−9^], are to be preferred to those in Table 1 when available.

Palmeri and Biemont [15] have measured 100 transitions from 4*f*, 5*g*, and 6*g* levels in Ar I, using spectra recorded with the same FTS used in the present experiment. In their hollow cathode source they used various Ar pressures but all were lower than ours; their average pressure of about 130 Pa (1 Torr) was lower than ours by a factor of 1/3. For the 4*f* → 3*d* transitions (66 lines), our wavenumbers agree beautifully with theirs; the mean deviation between the two sets of wavenumbers is 0.4(1.1) × 10^−3^ cm^−1^. For the 5*g* → 4*f* and 6*g* → 4*f* transitions (33 lines), our wavenumbers are, on average, greater than theirs by 6 × 10^−3^ cm^−1^. We attribute this difference to a pressure effect that appears to be strongly dependent on the angular momentum of the levels involved. This difference did not show up in our comparison with Norlén’s wavenumbers, even though his source pressure was lower than ours by a factor of 1/10, because the shift depends on the angular momentum of the levels involved and Norlén measured only transitions between *s*, *p*, and *d* orbitals in Ar I.

Any interaction between an excited Ar atom and neighboring atoms that shifts the energy of an excited level will also broaden it, and this effect is clearly seen in Table 1. For example, transitions near 3800 cm^−1^ from the 6*g* levels in Ar I have an average width ≈300 × 10^−3^ cm^−1^, while nearby lines from 4*d*′ and 5*p*′ upper levels have widths between (18 and 22) × 10^−3^ cm^−1^. The dependence on *l* appears to be greater than the dependence on *n*; nine lines from 7*p* configurations in the same (3600 to 4000) cm^−1^ range have an average width of 38 × 10^−3^ cm^−1^. In these examples, the lines are only broadened and shifted at the pressure (270 Pa to 530 Pa) of our source, but the lines may not be seen at all from a source at higher pressure. Palmeri and Biemont [15] report lines near 2100 cm^−1^ from 7*g* and 7*h* levels of Ar I that are broadened beyond recognition as lines on our spectra.

We conclude that the gas pressure in our hollow cathode source has *increased* the wavenumber of some lines from levels of high *l* or *n*. The shifted lines display a width noticeably greater than that of nearby transitions between *s*, *p*, and *d* levels. The wavenumbers, line widths, and relative signal amplitudes in the table are those one can expect to see from a hollow cathode discharge in Ar at a pressure of a few hundred Pa.

## Figures and Tables

**Table 1:** Ar I lines observed in the hollow cathode spectrum. The vacuum wavenumber (cm^−1^) in the first column has been converted to wavelength in standard air in the last column. Upper and lower levels are identified by the integer part of the level energy in cm^−1^, *J*-value (or [*K*]-value if individual *J* levels are not resolved), and configuration. Descriptive notes at the far right are as follows: B = blended line; R = reversed; ? = uncertain classification

Wavenumber	Unc.	log (*S*/*N*)	FWHM	Upper level^a^			Lower level^a^			Wavelength
(cm^−1^)	(cm^−1^)		(10^−3^ cm^−1^)	(cm^−1^)			(cm^−1^)			(nm)
1704.5679	.0008	1.10	21	121271	2	6*p*	119566	3	4*d*	5864.9902
1710.0644	.0005	1.23	18	121470	0	6*p*	119760	1	6*s*	5846.1389
1723.0187	.0003	2.23	15	118907	2	4*d*	117184	2	5*p*	5802.1855
1746.9721	.0004	1.36	17	121192	2	6*p*	119445	2	4*d*	5722.6295
1755.2831	.0003	1.71	15	118907	2	4*d*	117151	1	5*p*	5695.5337
1778.6263	.0003	1.45	19	122791	0	6*p*′	121012	1	4*d*′	5620.7838
1786.1520	.0016	0.84	22	122036	4	5*d*	120250	4	4*f*	5597.1015
1798.3588	.0003	1.77	14	116660	1	5*p*	114862	0	5*s*′	5559.1098
1806.8168	.0005	1.30	19	121655	2	4*f*′	119848	1	4*d*	5533.0867
1812.3256	.0003	2.26	19	121257	1	6*p*	119445	2	4*d*	5516.2681
1816.7271	.0005	1.18	15	117184	2	5*p*	115367	1	3*d*′	5502.9035
1825.7821	.0008	1.15	23	121271	2	6*p*	119445	2	4*d*	5475.6118
1828.9313	.0003	2.15	21	122036	4	5*d*	120207	5	4*f*	5466.1834
1850.9071	.0050	0.48	30	123505	2	5*d*′	121655	2	4*f*′	5401.2835
1852.2026	.0003	3.53	16	118512	0	4*d*	116660	1	5*p*	5397.5056
1853.5579	.0046	1.00	91	124136	3	5*f*′	122282	2	5*d*	5393.5590
1854.8601	.0003	2.15	15	116660	1	5*p*	114805	2	3*d*′	5389.7725
1856.8485	.0008	1.11	20	122087	2	5*d*	120230	2	4*f*	5384.0008
1857.1584	.0003	2.50	21	122087	2	5*d*	120230	3	4*f*	5383.1024
1881.5954	.0003	2.98	18	122635	2	6*p*′	120753	3	4*d*′	5313.1900
1898.2640	.0003	1.71	22	122087	2	5*d*	120189	2	4*f*	5266.5350
1898.6772	.0011	0.95	20	122087	2	5*d*	120188	1	4*f*	5265.3889
1903.1812	.0054	0.50	34	123557	3	5*d*′	121654	3	4*f*′	5252.9280
1904.1373	.0003	1.94	26	123557	3	5*d*′	121653	4	4*f*′	5250.2904
1907.2839	.0003	2.36	15	118907	2	4*d*	116999	2	5*p*	5241.6286
1910.2258	.0003	1.45	17	122160	3	5*d*	120250	4	4*f*	5233.5561
1910.2660	.0005	1.61	41	122160	3	5*d*	120250	3	4*f*	5233.4459
1930.0838	.0095	0.47	56	122160	3	5*d*	120230	2	4*f*	5179.7098
1930.3927	.0020	0.84	27	122160	3	5*d*	120230	3	4*f*	5178.8810
1942.2126	.0014	1.43	73	122696	4	5*f*	120753	3	4*d*′	5147.3634
1952.4926	.0003	2.51	19	121165	3	6*p*	119213	3	4*d*	5120.2621
1952.9706	.0003	2.48	23	122160	3	5*d*	120207	4	4*f*	5119.0089
1963.8546	.0003	1.77	16	118907	2	4*d*	116943	3	5*p*	5090.6386
1964.3675	.0030	1.30	121	122718	3	5*f*	120753	3	4*d*′	5089.3094
1971.1566	.0020	0.52	13	121654	3	4*f*′	119683	2	6*s*	5071.7807
1975.5468	.0043	0.95	76	124136	4	5*f*′	122160	3	5*d*	5060.5098
1978.9326	.0003	3.25	18	121192	2	6*p*	119213	3	4*d*	5051.8517
1990.6309	.0003	2.49	20	122610	1	6*p*′	120619	2	4*d*′	5022.1635
1991.3992	.0003	3.61	15	118651	1	4*d*	116660	1	5*p*	5020.2259
2000.3339	.0003	1.57	21	122601	1	6*p*′	120601	2	4*d*′	4997.8026
2007.1430	.0011	0.70	11	124715	3	7*d*	122708	3	5*f*	4980.8478
2010.6171	.0035	0.67	33	123172	1	7*p*	121161	1	6*s*′	4972.2416
2024.3074	.0003	1.75	15	116999	2	5*p*	114975	1	5*s*′	4938.6145
2029.2808	.0003	2.47	16	119213	3	4*d*	117184	2	5*p*	4926.5108
2032.2207	.0003	2.20	26	122282	2	5*d*	120250	3	4*f*	4919.3839
2034.1767	.0003	2.20	19	122635	2	6*p*′	120601	2	4*d*′	4914.6536
2050.2908	.0012	1.52	78	124137	3	5*f*′	122087	2	5*d*	4876.0273
2052.0339	.0004	1.50	26	122282	2	5*d*	120230	2	4*f*	4871.8853
2057.7439	.0003	1.49	21	121271	2	6*p*	119213	3	4*d*	4858.3664
2067.0419	.0054	0.66	49	122686	1	5*f*	120619	2	4*d*′	4836.5124
2067.3880	.0023	1.09	56	122686	2	5*f*	120619	2	4*d*′	4835.7027
2075.0395	.0004	1.13	11	125330	2	10*s*	123255	1	7*p*	4817.8716
2075.3327	.0029	0.67	27	123172	1	7*p*	121097	0	6*s*′	4817.1909
2079.7502	.0003	2.24	28	122330	3	5*d*	120250	4	4*f*	4806.9589
2080.8897	.0003	4.77	16	119024	4	4*d*	116943	3	5*p*	4804.3266
2085.5130	.0011	1.45	61	122686	2	5*f*	120601	2	4*d*′	4793.6761
2087.2321	.0003	1.73	18	121653	4	4*f*′	119566	3	4*d*	4789.7279
2088.1848	.0004	1.34	18	121654	3	4*f*′	119566	3	4*d*	4787.5427
2088.8664	.0012	1.56	86	122708	3	5*f*	120619	2	4*d*′	4785.9805
2089.1113	.0024	1.19	73	122708	2	5*f*	120619	2	4*d*′	4785.4194
2098.7920	.0051	1.21	164	122718	3	5*f*	120619	2	4*d*	4763.3466 B
2099.9107	.0004	1.53	28	122330	3	5*d*	120230	3	4*f*	4760.8090
2102.3065	.0003	1.71	31	123373	2	5*d*′	121271	2	6*p*	4755.3836
2106.9917	.0005	1.86	78	122708	3	5*f*	120601	2	4*d*′	4744.8093
2115.7642	.0024	0.78	29	123373	2	5*d*′	121257	1	6*p*	4725.1361
2120.5669	.0007	1.24	25	123774	3	6*d*	121653	4	4*f*′	4714.4345
2120.8171	.0003	3.14	15	116943	3	5*p*	114822	3	3*d*′	4713.8783
2122.4890	.0007	1.38	32	122330	3	5*d*	120207	4	4*f*	4710.1652
2131.8359	.0003	3.34	17	120601	2	4*d*′	118469	2	5*p*′	4689.5137
2140.9990	.0003	3.56	19	121012	1	4*d*′	118871	0	5*p*′	4669.4434
2141.2904	.0003	3.84	17	120601	2	4*d*′	118460	1	5*p*′	4668.8080
2141.7196	.0003	3.68	19	121165	3	6*p*	119024	4	4*d*	4667.8724
2149.9613	.0003	2.00	16	120619	2	4*d*′	118469	2	5*p*′	4649.9785
2153.0756	.0133	0.97	249	124871	4	7*g*	122718	4	5*f*	4643.2525
2154.2720	.0033	0.84	45	123809	2	6*d*	121654	3	4*f*′	4640.6738
2155.2844	.0005	1.16	15	123809	2	6*d*	121653	3	4*f*′	4638.4940
2159.4158	.0003	3.60	17	120619	2	4*d*′	118460	1	5*p*′	4629.6196
2160.9803	.0029	0.93	49	123816	1	5*d*′	121655	2	4*f*′	4626.2679
2162.1254	.0003	2.66	20	121069	1	6*p*	118907	2	4*d*	4623.8177
2171.5873	.0188	0.93	320	124867	5	7*g*	122696	5	5*f*	4603.6711
2176.3085	.0003	2.14	15	117151	1	5*p*	114975	1	5*s*′	4593.6841
2177.3877	.0003	2.82	15	116999	2	5*p*	114822	3	3*d*′	4591.4073
2193.4579	.0003	3.57	17	120601	2	4*d*′	118407	1	5*p*′	4557.7687
2194.1919	.0003	2.63	15	116999	2	5*p*	114805	2	3*d*′	4556.2440
2196.0919	.0003	1.84	16	117563	0	5*p*	115367	1	3*d*′	4552.3021
2196.7152	.0008	0.99	16	124357	2	8*p*	122160	3	5*d*	4551.0104
2197.2121	.0003	3.70	17	119760	1	6*s*	117563	0	5*p*	4549.9812
2204.5860	.0015	1.37	71	124137	2	5*f*′	121933	1	5*d*	4534.7624
2208.4057	.0003	2.65	18	121653	3	4*f*′	119445	2	4*d*	4526.9190
2208.5727	.0003	3.14	15	117184	2	5*p*	114975	1	5*s*′	4526.5767
2209.3987	.0005	1.25	18	121654	3	4*f*′	119445	2	4*d*	4524.8844
2209.7454	.0003	1.57	19	121655	2	4*f*′	119445	2	4*d*	4524.1744
2211.5834	.0003	4.03	17	120619	2	4*d*′	118407	1	5*p*′	4520.4145
2213.5460	.0003	4.46	16	119213	3	4*d*	116999	2	5*p*	4516.4065
2246.6156	.0003	3.99	16	118907	2	4*d*	116660	1	5*p*	4449.9264
2248.3218	.0011	0.95	19	123505	2	5*d*′	121257	1	6*p*	4446.5494
2251.7588	.0044	0.56	32	123509	0	6*d*	121257	1	6*p*	4439.7624
2258.7548	.0005	1.27	18	121165	3	6*p*	118907	2	4*d*	4426.0112
2261.2421	.0003	2.98	17	119445	2	4*d*	117184	2	5*p*	4421.1427
2270.1168	.0003	3.75	16	119213	3	4*d*	116943	3	5*p*	4403.8589
2284.4171	.0003	4.27	17	120753	3	4*d*′	118469	2	5*p*′	4376.2910
2284.8056	.0003	3.97	19	119848	1	4*d*	117563	0	5*p*	4375.5469
2285.1957	.0003	2.02	20	121192	2	6*p*	118907	2	4*d*	4374.7999
2289.6911	.0003	2.36	15	117151	1	5*p*	114862	0	5*s*′	4366.2108
2290.3931	.0003	2.40	17	121161	1	6*s*′	118871	0	5*p*′	4364.8726
2293.5068	.0003	4.18	17	119445	2	4*d*	117151	1	5*p*	4358.9467
2301.7641	.0003	1.98	16	116943	3	5*p*	114641	2	3*d*′	4343.3095
2312.9381	.0035	1.12	93	124349	3	8*p*	122036	4	5*d*	4322.3266
2313.6761	.0014	0.90	22	123505	2	5*d*′	121192	2	6*p*	4320.9479
2325.5328	.0009	1.32	38	122514	1	5*d*	120189	2	4*f*	4298.9176
2346.1926	.0003	2.48	16	117151	1	5*p*	114805	2	3*d*′	4261.0628
2350.5497	.0003	2.29	19	121257	1	6*p*	118907	2	4*d*	4253.1642
2358.3348	.0003	3.05	15	116999	2	5*p*	114641	2	3*d*′	4239.1241
2361.6531	.0003	2.94	16	117184	2	5*p*	114822	3	3*d*′	4233.1678
2364.0065	.0003	3.30	19	121271	2	6*p*	118907	2	4*d*	4228.9536
2365.6102	.0008	1.17	24	123557	3	5*d*′	121192	2	6*p*	4226.0867
2378.4572	.0003	3.68	16	117184	2	5*p*	114805	2	3*d*′	4203.2599
2382.4561	.0003	4.39	17	119566	3	4*d*	117184	2	5*p*	4196.2049
2392.0485	.0018	0.85	25	123557	3	5*d*′	121165	3	6*p*	4179.3776
2399.2345	.0008	1.33	33	123468	1	6*d*	121069	1	6*p*	4166.8598
2417.3418	.0003	2.91	20	121069	1	6*p*	118651	1	4*d*	4135.6476
2440.1867	.0015	1.02	31	123509	0	6*d*	121069	1	6*p*	4096.9299
2440.4078	.0003	2.96	18	121653	4	4*f*′	119213	3	4*d*	4096.5587
2441.3600	.0003	1.60	17	121654	3	4*f*′	119213	3	4*d*	4094.9609
2441.7057	.0018	0.71	18	121655	2	4*f*′	119213	3	4*d*	4094.3811
2445.5073	.0003	3.97	17	119445	2	4*d*	116999	2	5*p*	4088.0163
2459.5585	.0038	0.96	69	122709	[2.5]	5*g*	120250	4	4*f*	4064.6619 B
2459.5811	.0103	0.76	119	122709	[2.5]	5*g*	120250	3	4*f*	4064.6246 B
2465.6143	.0063	0.48	38	123936	1	8*s*	121470	0	6*p*	4054.6787
2469.5656	.0003	2.37	66	122719	[3.5]	5*g*	120250	4	4*f*	4048.1912 B
2469.5987	.0003	2.43	100	122719	[3.5]	5*g*	120250	3	4*f*	4048.1370 B
2473.0739	.0003	3.07	44	122723	[4.5]	5*g*	120250	4	4*f*	4042.4484 B
2473.1102	.0003	3.47	87	122723	4	5*g*	120250	3	4*f*	4042.3891
2479.4116	.0003	2.18	29	122709	[2.5]	5*g*	120230	2	4*f*	4032.1154
2479.7056	.0003	2.48	57	122709	[2.5]	5*g*	120230	3	4*f*	4031.6374 B
2487.7965	.0012	1.14	34	123653	4	6*d*	121165	3	6*p*	4018.5255
2489.4120	.0003	3.18	65	122719	3	5*g*	120230	2	4*f*	4015.9177
2489.7220	.0003	3.32	67	122719	[3.5]	5*g*	120230	3	4*f*	4015.4177 B
2493.9253	.0003	2.86	57	124149	3	5*g*	121655	2	4*f*	4008.6500
2494.2737	.0003	3.00	57	124148	[3.5]	5*g*	121654	3	4*f*	4008.0901 B
2495.0047	.0003	3.25	88	124148	[3.5]	5*g*	121653	3	4*f*	4006.9158 B
2495.2332	.0013	1.61	102	124148	[3.5]	5*g*	121653	4	4*f*	4006.5489 B
2499.4868	.0003	3.75	17	119683	2	6*s*	117184	2	5*p*	3999.7305
2502.0780	.0003	2.74	17	119445	2	4*d*	116943	3	5*p*	3995.5883
2505.6623	.0003	3.48	39	122713	[5.5]	5*g*	120207	4	4*f*	3989.8727 B
2505.7057	.0003	3.69	58	122713	[5.5]	5*g*	120207	5	4*f*	3989.8036 B
2510.3355	.0003	3.36	16	117151	1	5*p*	114641	2	3*d*′	3982.4453
2512.2643	.0003	1.76	18	116660	1	5*p*	114148	1	3*d*	3979.3877
2512.3413	.0048	0.98	92	122719	[3.5]	5*g*	120207	[4.5]	4*f*	3979.2658 B
2515.8301	.0003	2.19	78	122723	[4.5]	5*g*	120207	4	4*f*	3973.7475 B
2515.8629	.0003	2.26	109	122723	[4.5]	5*g*	120207	5	4*f*	3973.6957 B
2520.8112	.0003	3.31	56	122709	[2.5]	5*g*	120189	2	4*f*	3965.8955 B
2521.2210	.0003	3.08	60	122709	2	5*g*	120188	1	4*f*	3965.2508
2531.7513	.0003	2.90	17	119683	2	6*s*	117151	1	5*p*	3948.7582
2542.8637	.0003	3.20	19	121012	1	4*d*′	118469	2	5*p*′	3931.5020
2552.3181	.0003	2.94	19	121012	1	4*d*′	118460	1	5*p*′	3916.9387
2556.5390	.0003	2.56	21	121069	1	6*p*	118512	0	4*d*	3910.4718
2566.7213	.0003	3.55	17	119566	3	4*d*	116999	2	5*p*	3894.9588
2571.0392	.0136	0.44	75	123172	1	7*p*	120601	2	4*d*′	3888.4174
2576.5772	.0003	3.43	17	119760	1	6*s*	117184	2	5*p*	3880.0598
2587.9374	.0003	1.73	16	117563	0	5*p*	114975	1	5*s*′	3863.0276
2604.4858	.0003	2.76	19	121012	1	4*d*′	118407	1	5*p*′	3838.4827
2605.7661	.0003	2.19	20	121257	1	6*p*	118651	1	4*d*	3836.5967
2608.8419	.0003	3.80	17	119760	1	6*s*	117151	1	5*p*	3832.0734
2619.2228	.0003	2.42	20	121271	2	6*p*	118651	1	4*d*	3816.8855
2623.2920	.0003	3.86	18	119566	3	4*d*	116943	3	5*p*	3810.9648
2625.0351	.0014	1.00	28	123882	1	7*s*′	121257	1	6*p*	3808.4342
2632.5850	.0017	1.16	48	123903	2	8*s*	121271	2	6*p*	3797.5122
2637.0050	.0003	3.18	19	121097	0	6*s*′	118460	1	5*p*′	3791.1470
2664.1706	.0003	2.52	19	119848	1	4*d*	117184	2	5*p*	3752.4900
2665.2399	.0030	0.90	48	123936	1	8*s*	121271	2	6*p*	3750.9845
2678.6952	.0021	1.12	55	123936	1	8*s*	121257	1	6*p*	3732.1430
2683.7523	.0003	3.44	18	119683	2	6*s*	116999	2	5*p*	3725.1104
2689.1726	.0003	3.27	18	121097	0	6*s*′	118407	1	5*p*′	3717.6021
2690.3873	.0012	1.02	26	123882	1	7*s*′	121192	2	6*p*	3715.9236
2692.2580	.0003	3.86	18	121161	1	6*s*′	118469	2	5*p*′	3713.3416
2696.4354	.0003	4.11	19	119848	1	4*d*	117151	1	5*p*	3707.5888
2701.7123	.0003	3.65	18	121161	1	6*s*′	118460	1	5*p*′	3700.3473
2711.4036	.0003	1.98	15	123903	2	8*s*	121192	2	6*p*	3687.1212 B
2725.0243	.0067	0.65	60	125335	0	8*s*′	122610	1	6*p*′	3668.6916
2737.8368	.0006	1.59	44	123903	2	8*s*	121165	3	6*p*	3651.5229
2740.3226	.0003	4.28	18	119683	2	6*s*	116943	3	5*p*	3648.2105
2744.0525	.0009	1.37	40	123936	1	8*s*	121192	2	6*p*	3643.2516
2744.9632	.0003	1.72	21	121257	1	6*p*	118512	0	4*d*	3642.0429
2747.6223	.0003	3.57	18	121654	3	4*f*′	118907	2	4*d*	3638.5182
2747.9691	.0003	2.44	18	121655	2	4*f*′	118907	2	4*d*	3638.0590
2753.4613	.0026	0.70	26	122601	1	6*p*′	119848	1	4*d*	3630.8023
2753.8798	.0003	3.82	18	121161	1	6*s*′	118407	1	5*p*′	3630.2505
2760.8424	.0003	4.08	18	119760	1	6*s*	116999	2	5*p*	3621.0954
2784.8409	.0012	0.85	17	119445	2	4*d*	116660	1	5*p*	3589.8904
2813.4539	.0039	0.60	31	123882	1	7*s*′	121069	1	6*p*	3553.3810
2818.8415	.0003	2.03	20	121470	0	6*p*	118651	1	4*d*	3546.5895
2834.4679	.0017	1.19	54	123903	2	8*s*	121069	1	6*p*	3527.0371
2838.2967	.0023	1.25	83	122686	1	5*f*	119848	1	4*d*	3522.2792
2838.6384	.0003	2.52	58	122686	2	5*f*	119848	1	4*d*	3521.8552
2848.4359	.0003	2.15	20	119848	1	4*d*	116999	2	5*p*	3509.7414
2851.5955	.0003	2.28	17	116999	2	5*p*	114148	1	3*d*	3505.8526
2860.3627	.0003	2.43	81	122708	2	5*f*	119848	1	4*d*	3495.1069
2874.8957	.0039	0.64	34	122635	2	6*p*′	119760	1	6*s*	3477.4387
2918.1446	.0007	1.17	21	122601	1	6*p*′	119683	2	6*s*	3425.9006
2926.5652	.0036	0.69	35	122610	1	6*p*′	119683	2	6*s*	3416.0433
2942.7840	.0072	0.59	56	122791	0	6*p*′	119848	1	4*d*	3397.2162
2947.9582	.0023	1.30	90	122708	2	5*f*	119760	1	6*s*	3391.2534
2976.1176	.0023	1.23	78	124137	2	5*f*′	121161	1	6*s*′	3359.1661
3003.1852	.0003	2.94	19	121655	2	4*f*′	118651	1	4*d*	3328.8900
3003.5961	.0003	4.16	17	117151	1	5*p*	114148	1	3*d*	3328.4346
3016.7344	.0003	4.10	16	116660	1	5*p*	113643	1	5*s*	3313.9388
3023.0838	.0003	3.94	18	119683	2	6*s*	116660	1	5*p*	3306.9785
3024.8017	.0022	1.32	90	122708	3	5*f*	119683	2	6*s*	3305.1004
3035.8606	.0003	2.43	17	117184	2	5*p*	114148	1	3*d*	3293.0607
3040.5660	.0003	3.57	18	118407	1	5*p*′	115367	1	3*d*′	3287.9646
3061.9268	.0003	2.51	21	121933	1	5*d*	118871	0	5*p*′	3265.0269
3069.0179	.0011	1.04	25	122635	2	6*p*′	119566	3	4*d*	3257.4829
3092.7336	.0003	3.57	18	118460	1	5*p*′	115367	1	3*d*′	3232.5038
3095.4093	.0003	3.01	15	111818	1	3*d*	108723	0	4*p*′	3229.7096
3100.1742	.0003	3.25	18	119760	1	6*s*	116660	1	5*p*	3224.7456
3102.1880	.0003	2.81	18	118469	2	5*p*′	115367	1	3*d*′	3222.6523
3120.3530	.0033	1.00	65	122686	2	5*f*	119566	3	4*d*	3203.8917
3125.5136	.0004	1.96	74	124137	2	5*f*′	121012	1	4*d*′	3198.6017
3129.6340	.0003	2.05	61	122696	4	5*f*	119566	3	4*d*	3194.3905
3141.8337	.0004	2.03	75	122708	3	5*f*	119566	3	4*d*	3181.9867
3143.1718	.0013	1.08	32	123373	2	5*d*′	120230	3	4*f*	3180.6321
3151.7997	.0003	2.60	92	122718	4	5*f*	119566	3	4*d*	3171.9253
3164.8110	.0006	1.25	22	122610	1	6*p*′	119445	2	4*d*	3158.8847
3187.7678	.0003	2.01	20	119848	1	4*d*	116660	1	5*p*	3136.1359
3191.5209	.0003	4.70	17	116660	1	5*p*	113468	2	5*s*	3132.4479
3226.2014	.0003	3.64	17	116943	3	5*p*	113717	3	3*d*	3098.7752
3234.0312	.0003	2.09	24	116660	1	5*p*	113426	2	3*d*	3091.2729 B
3255.6064	.0111	0.43	60	123505	2	5*d*′	120250	3	4*f*	3070.7867
3263.2924	.0006	1.81	78	122708	2	5*f*	119445	2	4*d*	3063.5541
3272.9837	.0003	2.40	91	122718	3	5*f*	119445	2	4*d*	3054.4830
3279.3267	.0039	0.90	62	123468	1	6*d*	120189	2	4*f*	3048.5749
3279.7342	.0042	0.70	42	123468	1	6*d*	120188	1	4*f*	3048.1961
3282.7720	.0003	3.49	17	116999	2	5*p*	113717	3	3*d*	3045.3753
3320.6895	.0044	0.71	45	123509	0	6*d*	120188	1	4*f*	3010.6015
3334.4971	.0003	1.91	21	121794	0	5*d*	118460	1	5*p*′	2998.1351
3350.2442	.0019	0.94	33	123557	3	5*d*′	120207	4	4*f*	2984.0430
3356.0661	.0003	4.96	17	116999	2	5*p*	113643	1	5*s*	2978.8665
3382.2227	.0003	2.34	68	124136	4	5*f*′	120753	3	4*d*′	2955.8292
3383.7324	.0044	0.95	78	124137	3	5*f*′	120753	3	4*d*′	2954.5105
3386.6647	.0009	1.09	23	121794	0	5*d*	118407	1	5*p*′	2951.9523
3403.2640	.0077	0.53	52	123653	4	6*d*	120250	4	4*f*	2937.5543
3407.1346	.0013	1.13	35	123255	1	7*p*	119848	1	4*d*	2934.2171
3415.2255	.0003	3.77	18	117563	0	5*p*	114148	1	3*d*	2927.2658
3417.2967	.0003	3.43	19	120601	2	4*d*′	117184	2	5*p*	2925.4916
3422.1922	.0006	1.31	22	122635	2	6*p*′	119213	3	4*d*	2921.3066
3432.4115	.0003	4.12	17	118407	1	5*p*′	114975	1	5*s*′	2912.6090
3435.4222	.0003	3.18	19	120619	2	4*d*′	117184	2	5*p*	2910.0565
3444.4688	.0107	0.60	85	124610	4	7*d*	121165	3	6*p*	2902.4135
3446.0344	.0004	1.67	36	123653	4	6*d*	120207	5	4*f*	2901.0949
3448.9604	.0009	1.07	22	121012	1	4*d*′	117563	0	5*p*	2898.6337
3449.5614	.0003	2.95	19	120601	2	4*d*′	117151	1	5*p*	2898.1286
3460.5026	.0006	1.39	31	123221	2	7*p*	119760	1	6*s*	2888.9655
3463.7917	.0003	2.11	22	121933	1	5*d*	118469	2	5*p*′	2886.2222
3467.0373	.0003	4.35	18	117184	2	5*p*	113717	3	3*d*	2883.5204
3467.6864	.0003	1.57	20	120619	2	4*d*′	117151	1	5*p*	2882.9806
3474.2819	.0064	0.12	17	116943	3	5*p*	113468	2	5*s*	2877.5076
3482.0739	.0031	1.00	62	124643	1	7*p*′	121161	1	6*s*′	2871.0685
3482.8101	.0003	2.74	60	122696	4	5*f*	119213	3	4*d*	2870.4616
3484.5791	.0003	4.10	18	118460	1	5*p*′	114975	1	5*s*′	2869.0044
3488.8505	.0008	1.36	35	123172	1	7*p*	119683	2	6*s*	2865.4918
3494.0335	.0003	4.72	18	118469	2	5*p*′	114975	1	5*s*′	2861.2412
3494.7287	.0008	1.25	27	123255	1	7*p*	119760	1	6*s*	2860.6720
3497.1292	.0020	0.97	38	124658	2	7*p*′	121161	1	6*s*′	2858.7084
3501.3603	.0043	0.80	54	123262	2	7*p*	119760	1	6*s*	2855.2539
3504.0525	.0003	3.47	19	118871	0	5*p*′	115367	1	3*d*′	2853.0602
3504.9537	.0005	2.04	103	122718	3	5*f*	119213	3	4*d*	2852.3266
3508.0666	.0003	4.81	18	117151	1	5*p*	113643	1	5*s*	2849.7956
3516.6474	.0003	2.05	67	124136	3	5*f*′	120619	2	4*d*′	2842.8419
3518.1904	.0008	1.67	69	124137	3	5*f*′	120619	2	4*d*′	2841.5951
3522.7069	.0004	1.63	35	123206	3	7*p*	119683	2	6*s*	2837.9519
3525.4137	.0003	2.05	23	121933	1	5*d*	118407	1	5*p*′	2835.7729
3530.8526	.0003	4.34	17	116999	2	5*p*	113468	2	5*s*	2831.4047
3534.7731	.0005	1.87	69	124136	3	5*f*′	120601	2	4*d*′	2828.2643
3536.3163	.0003	2.09	73	124137	3	5*f*′	120601	2	4*d*′	2827.0301
3536.5335	.0048	1.25	172	124137	2	5*f*′	120601	2	4*d*′	2826.8565
3537.2741	.0017	1.20	55	123385	0	7*p*	119848	1	4*d*	2826.2646
3537.5832	.0033	1.00	65	123221	2	7*p*	119683	2	6*s*	2826.0176
3540.3313	.0003	4.46	18	117184	2	5*p*	113643	1	5*s*	2823.8240
3545.7945	.0003	4.27	18	118407	1	5*p*′	114862	0	5*s*′	2819.4732
3566.6722	.0026	1.07	61	123774	3	6*d*	120207	4	4*f*	2802.9693
3569.8779	.0003	1.88	19	120753	3	4*d*′	117184	2	5*p*	2800.4522
3573.3636	.0003	4.12	18	116999	2	5*p*	113426	2	3*d*	2797.7205
3576.8590	.0039	0.83	53	123827	2	6*d*	120250	3	4*f*	2794.9865
3578.4471	.0005	1.56	35	123809	2	6*d*	120230	2	4*f*	2793.7461
3578.7552	.0026	0.95	47	123809	2	6*d*	120230	3	4*f*	2793.5055
3582.4919	.0032	0.97	60	123832	3	6*d*	120250	4	4*f*	2790.5918
3597.9620	.0003	4.18	18	118460	1	5*p*′	114862	0	5*s*′	2778.5931
3598.3539	.0003	1.81	19	121161	1	6*s*′	117563	0	5*p*	2778.2905
3602.2960	.0003	2.17	18	118407	1	5*p*′	114805	2	3*d*′	2775.2501
3617.8589	.0003	2.18	24	122087	2	5*d*	118469	2	5*p*′	2763.3119
3619.6875	.0003	2.70	19	120619	2	4*d*′	116999	2	5*p*	2761.9159
3624.8701	.0008	1.42	40	123385	0	7*p*	119760	1	6*s*	2757.9671
3627.3130	.0003	2.56	24	122087	2	5*d*	118460	1	5*p*′	2756.1097
3639.7370	.0016	1.05	36	123206	3	7*p*	119566	3	4*d*	2746.7019
3643.2630	.0039	0.84	54	122514	1	5*d*	118871	0	5*p*′	2744.0436
3647.1139	.0003	4.30	18	118469	2	5*p*′	114822	3	3*d*′	2741.1462
3654.4636	.0003	4.01	18	118460	1	5*p*′	114805	2	3*d*′	2735.6333
3658.1322	.0006	1.18	19	120601	2	4*d*′	116943	3	5*p*	2732.8899
3663.9180	.0003	3.01	18	118469	2	5*p*′	114805	2	3*d*′	2728.5743
3671.9998	.0003	2.86	61	122696	5	5*f*	119024	4	4*d*	2722.5689
3676.2657	.0003	1.96	37	120619	2	4*d*′	116943	3	5*p*	2719.4096
3682.8532	.0003	3.98	18	117151	1	5*p*	113468	2	5*s*	2714.5454
3691.0920	.0003	2.10	25	122160	3	5*d*	118469	2	5*p*′	2708.4864
3694.2020	.0010	1.69	94	122718	[3.5]	5*f*	119024	4	4*d*	2706.2062 B
3694.6143	.0003	1.77	24	122601	1	6*p*′	118907	2	4*d*	2705.9042
3695.4821	.0005	1.68	43	123262	2	7*p*	119566	3	4*d*	2705.2688
3703.0354	.0007	1.21	21	122610	1	6*p*′	118907	2	4*d*	2699.7507
3715.1178	.0003	4.79	18	117184	2	5*p*	113468	2	5*s*	2690.9705
3725.3642	.0003	4.17	18	117151	1	5*p*	113426	2	3*d*	2683.5691
3728.4570	.0015	0.88	23	122635	2	6*p*′	118907	2	4*d*	2681.3431
3737.8865	.0028	0.91	45	124750	0	7*p*′	121012	1	4*d*′	2674.5789
3754.1430	.0003	2.60	19	120753	3	4*d*′	116999	2	5*p*	2662.9973
3757.6289	.0003	3.31	18	117184	2	5*p*	113426	2	3*d*	2660.5268
3766.4389	.0003	4.12	18	118407	1	5*p*′	114641	2	3*d*′	2654.3036
3775.8378	.0007	1.34	32	123221	2	7*p*	119445	2	4*d*	2647.6965
3779.4472	.0047	0.87	69	122686	1	5*f*	118907	2	4*d*	2645.1679
3779.7932	.0003	2.04	57	122686	2	5*f*	118907	2	4*d*	2644.9258
3801.2718	.0003	2.27	76	122708	3	5*f*	118907	2	4*d*	2629.9810
3810.0638	.0005	1.59	38	123255	1	7*p*	119445	2	4*d*	2623.9121
3810.6389	.0036	0.74	40	124061	[3.5]	6*g*	120250	[3.5]	4*f*	2623.5161 B
3810.7137	.0003	2.90	20	120753	3	4*d*′	116943	3	5*p*	2623.4646
3812.7985	.0051	1.24	178	124063	[4.5]	6*g*	120250	[3.5]	4*f*	2622.0301 B
3818.6059	.0008	1.13	20	118460	1	5*p*′	114641	2	3*d*′	2618.0425
3825.0158	.0126	1.19	389	124055	[2.5]	6*g*	120230	2	4*f*	2613.6552 B
3825.3040	.0059	1.25	209	124055	[2.5]	6*g*	120230	3	4*f*	2613.4578 B
3828.0610	.0003	3.28	19	118469	2	5*p*′	114641	2	3*d*′	2611.5760
3828.3245	.0003	2.83	22	121012	1	4*d*′	117184	2	5*p*	2611.3963
3830.5014	.0106	1.21	343	124061	3	6*g*	120230	2	4*f*	2609.9122
3830.7906	.0018	1.75	200	124061	[3.5]	6*g*	120230	3	4*f*	2609.7152 B
3836.2337	.0144	1.05	324	125491	[3.5]	6*g*′	121655	2	4*f*′	2606.0123
3836.6027	.0062	1.25	222	125491	[3.5]	6*g*′	121654	3	4*f*′	2605.7617 B
3837.4857	.0311	0.62	259	125491	[4.5]	6*g*′	121653	[3.5]	4*f*′	2605.1621 B
3849.8042	.0008	2.29	307	124057	[5.5]	6*g*	120207	[4.5]	4*f*	2596.8262 B
3855.4906	.0165	0.97	307	124063	[4.5]	6*g*	120207	[4.5]	4*f*	2592.9962 B
3860.6123	.0009	1.18	26	122330	3	5*d*	118469	2	5*p*′	2589.5562
3866.4002	.0026	1.76	294	124055	[2.5]	6*g*	120189	2	4*f*	2585.6797 B
3866.8038	.0036	1.61	294	124055	2	6*g*	120188	1	4*f*	2585.4098
3874.6649	.0003	1.79	29	122282	2	5*d*	118407	1	5*p*′	2580.1644
3895.8981	.0003	4.28	18	118871	0	5*p*′	114975	1	5*s*′	2566.1021
3904.9717	.0006	1.49	36	124658	2	7*p*′	120753	3	4*d*′	2560.1395
3919.6961	.0003	4.43	18	117563	0	5*p*	113643	1	5*s*	2550.5223
3922.4009	.0003	3.73	19	116943	3	5*p*	113020	3	3*d*	2548.7635
3940.8940	.0003	1.93	20	120601	2	4*d*′	116660	1	5*p*	2536.8031
3945.2764	.0003	1.67	21	121097	0	6*s*′	117151	1	5*p*	2533.9852
3959.0191	.0003	1.68	20	120619	2	4*d*′	116660	1	5*p*	2525.1892
3977.7190	.0007	1.20	21	121161	1	6*s*′	117184	2	5*p*	2513.3178
3978.9716	.0003	4.69	19	116999	2	5*p*	113020	3	3*d*	2512.5266
3983.6714	.0007	1.28	24	122635	2	6*p*′	118651	1	4*d*	2509.5624
3992.9132	.0013	1.20	42	123206	3	7*p*	119213	3	4*d*	2503.7539
4007.7998	.0003	1.91	34	123221	2	7*p*	119213	3	4*d*	2494.4540
4012.5897	.0003	1.57	23	121012	1	4*d*′	116999	2	5*p*	2491.4763
4024.3750	.0012	1.20	38	124643	1	7*p*′	120619	2	4*d*′	2484.1800
4034.6659	.0005	1.74	55	122686	1	5*f*	118651	1	4*d*	2477.8438
4035.0096	.0003	2.12	57	122686	2	5*f*	118651	1	4*d*	2477.6328
4042.4994	.0017	1.08	40	124643	1	7*p*′	120601	2	4*d*′	2473.0423
4048.6680	.0021	0.78	25	123262	2	7*p*	119213	3	4*d*	2469.2743
4054.5812	.0015	1.15	42	122514	1	5*d*	118460	1	5*p*′	2465.6732
4056.7341	.0018	1.35	79	122708	2	5*f*	118651	1	4*d*	2464.3646
4097.4490	.0028	0.67	26	122610	1	6*p*′	118512	0	4*d*	2439.8771
4106.7486	.0028	0.87	42	122514	1	5*d*	118407	1	5*p*′	2434.3521
4139.1473	.0013	0.98	24	122791	0	6*p*′	118651	1	4*d*	2415.2974
4161.9827	.0018	0.79	22	121161	1	6*s*′	116999	2	5*p*	2402.0455
4163.2368	.0003	2.73	19	117184	2	5*p*	113020	3	3*d*	2401.3219
4171.3499	.0003	4.34	18	111668	0	3*d*	107496	1	4*p*′	2396.6515
4173.8640	.0005	1.83	57	122686	1	5*f*	118512	0	4*d*	2395.2079
4182.1415	.0003	2.01	36	123206	3	7*p*	119024	4	4*d*	2390.4671
4192.6019	.0003	4.81	19	116943	3	5*p*	112750	4	3*d*	2384.5030
4193.5551	.0068	1.15	192	124041	2	6*f*	119848	1	4*d*	2383.9610
4203.8383	.0075	1.14	206	124052	2	6*f*	119848	1	4*d*	2378.1295
4259.7018	.0049	0.49	30	118407	1	5*p*′	114148	1	3*d*	2346.9417
4265.3208	.0023	0.97	42	123172	1	7*p*	118907	2	4*d*	2343.8499
4311.8672	.0003	1.82	21	118460	1	5*p*′	114148	1	3*d*	2318.5481
4321.6119	.0003	4.28	18	111818	1	3*d*	107496	1	4*p*′	2313.3201
4348.2926	.0052	0.69	51	123255	1	7*p*	118907	2	4*d*	2299.1258
4351.9215	.0003	2.82	23	121012	1	4*d*′	116660	1	5*p*	2297.2086
4354.9209	.0005	1.65	45	123262	2	7*p*	118907	2	4*d*	2295.6264
4369.8870	.0003	1.75	25	121933	1	5*d*	117563	0	5*p*	2287.7643
4436.6086	.0003	2.07	22	121097	0	6*s*′	116660	1	5*p*	2253.3589
4471.3904	.0205	0.42	108	125483	2	6*f*′	121012	1	4*d*′	2235.8307
4480.5603	.0185	0.73	199	124047	4	6*f*	119566	3	4*d*	2231.2458
4485.2981	.0166	0.79	205	124051	3	6*f*	119566	3	4*d*	2228.8980
4492.1761	.0055	1.31	226	124058	4	6*f*	119566	3	4*d*	2225.4853
4501.3157	.0003	1.92	22	121161	1	6*s*′	116660	1	5*p*	2220.9666
4520.5363	.0012	1.22	41	123172	1	7*p*	118651	1	4*d*	2211.5234
4521.0712	.0003	3.38	20	116660	1	5*p*	112139	2	3*d*	2211.2617
4528.3284	.0003	4.26	19	111818	1	3*d*	107290	2	4*p*′	2207.7179
4536.0578	.0003	3.65	19	111668	0	3*d*	107132	1	4*p*′	2203.9560
4597.0516	.0042	0.59	33	123468	1	6*d*	118871	0	5*p*′	2174.7138
4606.7673	.0388	0.54	269	124052	2	6*f*	119445	2	4*d*	2170.1273
4621.1713	.1252	0.58	952	124871	[4.5]	7*g*	120250	[3.5]	4*f*	2163.3631 B
4639.8400	.1223	0.43	658	124870	[3.5]	7*g*	120230	[2.5]	4*f*	2154.6587 B
4642.5082	.0003	4.19	19	112139	2	3*d*	107496	1	4*p*′	2153.4203
4659.7338	.0060	0.77	71	123172	1	7*p*	118512	0	4*d*	2145.4598
4660.0886	.0303	0.80	382	124867	[5.5]	7*g*	120207	[4.5]	4*f*	2145.2964 B
4660.1418	.0150	0.80	189	124867	[5.5]	7*g*	120207	[4.5]	4*f*	2145.2719 B
4677.3691	.1915	0.25	681	124866	[2.5]	7*g*	120189	2	4*f*	2137.3707 B
4677.8035	.0695	0.10	175	124866	2	7*g*	120188	1	4*f*	2137.1722
4686.3198	.0003	3.48	19	111818	1	3*d*	107132	1	4*p*′	2133.2883
4723.1855	.0006	1.28	21	118871	0	5*p*′	114148	1	3*d*	2116.6375
4729.1871	.0045	1.08	109	125483	3	6*f*′	120753	3	4*d*′	2113.9513
4749.2522	.0003	1.67	26	121933	1	5*d*	117184	2	5*p*	2105.0201
4752.4982	.0003	2.68	21	118469	2	5*p*′	113717	3	3*d*	2103.5823
4763.7568	.0003	4.41	20	111818	1	3*d*	107054	0	4*p*	2098.6108
4781.5173	.0013	0.98	24	121933	1	5*d*	117151	1	5*p*	2090.8157
4803.8323	.0003	3.39	21	116943	3	5*p*	112139	2	3*d*	2081.1033
4821.3722	.0003	2.75	23	120188	1	4*f*	115367	1	3*d*′	2073.3556
4821.7833	.0003	3.37	23	120189	2	4*f*	115367	1	3*d*′	2073.3556
4825.7921	.0003	2.64	21	118469	2	5*p*′	113643	1	5*s*	2071.6332
4833.7383	.0043	1.32	179	124047	4	6*f*	119213	3	4*d*	2068.2276
4841.9676	.0003	3.60	21	116660	1	5*p*	111818	1	3*d*	2064.7125
4845.3393	.0496	0.42	261	124058	4	6*f*	119213	3	4*d*	2063.2758
4849.2246	.0003	4.64	20	112139	2	3*d*	107290	2	4*p*′	2061.6226
4860.4029	.0003	3.09	21	116999	2	5*p*	112139	2	3*d*	2056.8812
4863.1981	.0003	1.91	24	120230	2	4*f*	115367	1	3*d*′	2055.6989
4863.6156	.0498	0.52	330	125483	3	6*f*	120619	2	4*d*	2055.5225
4881.7088	.0738	0.20	234	125483	3	6*f*′	120601	2	4*d*′	2047.9040
4882.2183	.0619	0.39	304	125483	3	6*f*′	120601	2	4*d*′	2047.6903
4903.3195	.0005	1.44	27	122087	2	5*d*	117184	2	5*p*	2038.8782
4903.8743	.0022	0.90	34	123373	2	5*d*′	118469	2	5*p*′	2038.6475
4913.3272	.0010	1.29	37	123373	2	5*d*′	118460	1	5*p*′	2034.7253
4916.4170	.0006	1.46	34	122479	1	7*s*	117563	0	5*p*	2033.4465
4920.6418	.0003	3.75	21	113643	1	5*s*	108723	0	4*p*′	2031.7006
4933.5165	.0019	0.88	28	121933	1	5*d*	116999	2	5*p*	2026.3986
4935.5840	.0013	0.84	18	122087	2	5*d*	117151	1	5*p*	2025.5498
4938.9606	.0019	0.71	19	118407	1	5*p*′	113468	2	5*s*	2024.1650
4981.4677	.0003	2.88	22	118407	1	5*p*′	113426	2	3*d*	2006.8927
4991.1242	.0003	2.78	22	118460	1	5*p*′	113468	2	5*s*	2003.0098
4992.2295	.0003	3.21	22	116660	1	5*p*	111668	0	3*d*	2002.5664
5000.5785	.0003	2.12	22	118469	2	5*p*′	113468	2	5*s*	1999.2229
5007.2161	.0003	3.89	20	112139	2	3*d*	107132	1	4*p*′	1996.5727
5011.2612	.0069	0.43	37	123882	1	7*s*′	118871	0	5*p*′	1994.9610
5012.4035	.0003	3.09	22	117151	1	5*p*	112139	2	3*d*	1994.5064
5022.9444	.0055	1.19	171	124047	5	6*f*	119024	4	4*d*	1990.3208
5033.6355	.0004	1.57	22	118460	1	5*p*′	113426	2	3*d*	1986.0935
5043.0896	.0003	1.65	22	118469	2	5*p*′	113426	2	3*d*	1982.3703
5044.6682	.0003	4.11	22	117184	2	5*p*	112139	2	3*d*	1981.7499
5088.3662	.0024	0.76	27	123557	3	5*d*′	118469	2	5*p*′	1964.7310
5093.3095	.0003	2.01	28	122036	4	5*d*	116943	3	5*p*	1962.8241
5098.0554	.0013	1.03	28	123505	2	5′	118407	1	5*p*′	1960.9969
5134.1000	.0004	1.60	27	121794	0	5*d*	116660	1	5*p*	1947.2294
5134.7213	.0111	0.50	70							1946.9938
5143.9814	.0103	0.50	65							1943.4889
5144.1578	.0041	0.63	35	122087	2	5*d*	116943	3	5*p*	1943.4222
5144.7379	.0185	0.75	208	124051	3	6*f*	118907	2	4*d*	1943.2031
5160.8178	.0014	1.02	30	122160	3	5*d*	116999	2	5*p*	1937.1485
5181.2992	.0003	1.82	22	116999	2	5*p*	111818	1	3*d*	1929.4911
5213.2166	.0003	1.98	23	120188	1	4*f*	114975	1	5*s*′	1917.6779
5213.6287	.0003	2.34	24	120189	2	4*f*	114975	1	5*s*′	1917.5264
5227.6566	.0003	2.06	22	118871	0	5*p*′	113643	1	5*s*	1912.3809
5255.0438	.0008	1.18	24	120230	2	4*f*	114975	1	5*s*′	1902.4143
5256.4671	.0005	1.54	32	122440	2	7*s*	117184	2	5*p*	1901.8992
5288.7302	.0078	0.59	61	122440	2	7*s*	117151	1	5*p*	1890.2969
5295.7768	.0013	1.27	48	122479	1	7*s*	117184	2	5*p*	1887.7817
5325.3649	.0005	1.43	27	124349	3	8*p*	119024	4	4*d*	1877.2930
5326.5996	.0003	1.86	28	120188	1	4*f*	114862	0	5*s*′	1876.8579
5328.0407	.0008	1.47	43	122479	1	7*s*	117151	1	5*p*	1876.3502
5333.2985	.0003	2.75	26	117151	1	5*p*	111818	1	3*d*	1874.5005
5362.8422	.0017	1.30	69	122514	1	5*d*	117151	1	5*p*	1864.1739
5365.5629	.0003	3.03	26	117184	2	5*p*	111818	1	3*d*	1863.2286
5366.7097	.0005	1.50	27	120189	2	4*f*	114822	3	3*d*′	1862.8305
5383.1008	.0003	2.19	27	120188	1	4*f*	114805	2	3*d*′	1857.1583
5383.5132	.0003	3.06	28	120189	2	4*f*	114805	2	3*d*′	1857.0160
5385.2359	.0003	2.86	28	120207	4	4*f*	114822	3	3*d*′	1856.4220
5407.8144	.0003	2.69	28	120230	3	4*f*	114822	3	3*d*′	1848.6711
5408.1245	.0003	1.74	30	120230	2	4*f*	114822	3	3*d*′	1848.5651
5413.1355	.0010	1.40	50	123882	1	7*s*′	118469	2	5*p*′	1846.8539
5413.3861	.0029	0.96	52	123873	0	7*s*′	118460	1	5*p*′	1846.7684
5422.5872	.0031	0.93	52	123882	1	7*s*′	118460	1	5*p*′	1843.6348
5424.6185	.0003	3.57	28	120230	3	4*f*	114805	2	3*d*′	1842.9444
5424.9288	.0003	2.67	29	120230	2	4*f*	114805	2	3*d*′	1842.8390
5425.1122	.0003	3.97	25	114148	1	3*d*	108723	0	4*p*′	1842.7767
5426.9133	.0012	1.22	39	122087	2	5*d*	116660	1	5*p*	1842.1651
5427.9369	.0003	2.47	27	120250	3	4*f*	114822	3	3*d*′	1841.8177
5427.9759	.0003	3.22	29	120250	4	4*f*	114822	3	3*d*′	1841.8045
5440.7264	.0013	1.24	45	122440	2	7*s*	116999	2	5*p*	1837.4881
5444.7413	.0003	1.92	28	120250	3	4*f*	114805	2	3*d*′	1836.1332
5448.6962	.0003	2.34	27	118469	2	5*p*′	113020	3	3*d*	1834.8005
5465.5582	.0017	1.06	39	123873	0	7*s*′	118407	1	5*p*′	1829.1398
5474.7706	.0003	1.96	31	123882	1	7*s*′	118407	1	5*p*′	1826.0619
5480.0418	.0004	1.86	44	122479	1	7*s*	116999	2	5*p*	1824.3055
5483.5605	.0003	2.20	26	117151	1	5*p*	111668	0	3*d*	1823.1348
5497.2964	.0003	2.08	44	122440	2	7*s*	116943	3	5*p*	1818.5794
5547.2429	.0006	1.41	27	120188	1	4*f*	114641	2	3*d*′	1802.2052
5547.6563	.0004	1.60	29	120189	2	4*f*	114641	2	3*d*′	1802.0709
5580.4771	.0003	4.46	25	111818	1	3*d*	106238	2	4*p*	1791.4723
5580.5079	.0003	2.47	20	111668	0	3*d*	106087	1	4*p*	1791.4624
5588.7614	.0003	2.38	29	120230	3	4*f*	114641	2	3*d*′	1788.8167
5589.0716	.0003	2.85	30	120230	2	4*f*	114641	2	3*d*′	1788.7175
5608.8840	.0003	3.51	29	120250	3	4*f*	114641	2	3*d*′	1782.3991
5730.6554	.0003	2.93	25	113020	3	3*d*	107290	2	4*p*′	1744.5247
5730.7691	.0003	3.79	25	111818	1	3*d*	106087	1	4*p*	1744.4901
5744.9275	.0003	2.90	28	117563	0	5*p*	111818	1	3*d*	1740.1907
5780.0583	.0004	1.80	45	122440	2	7*s*	116660	1	5*p*	1729.6140
5819.3713	.0022	1.09	53	122479	1	7*s*	116660	1	5*p*	1717.9295
5901.3725	.0003	5.09	26	112139	2	3*d*	106238	2	4*p*	1694.0584
5929.5467	.0003	2.22	26	113426	2	3*d*	107496	1	4*p*′	1686.0090
5972.0567	.0003	3.92	27	113468	2	5*s*	107496	1	4*p*′	1674.0078
6040.5042	.0003	2.79	31	120188	1	4*f*	114148	1	3*d*	1655.0388
6040.9167	.0003	3.72	32	120189	2	4*f*	114148	1	3*d*	1654.9258
6051.6654	.0003	4.19	27	112139	2	3*d*	106087	1	4*p*	1651.9864
6082.3322	.0003	4.02	33	120230	2	4*f*	114148	1	3*d*	1643.6572
6146.8432	.0003	2.85	28	113643	1	5*s*	107496	1	4*p*′	1626.4070
6178.7732	.0003	3.56	28	113468	2	5*s*	107290	2	4*p*′	1618.0022
6189.3304	.0010	1.49	63	123373	2	5*d*′	117184	2	5*p*	1615.2424
6200.7575	.0003	3.17	27	111818	1	3*d*	105617	2	4*p*	1612.2657
6252.3992	.0003	4.30	29	114975	1	5*s*′	108723	0	4*p*′	1598.9492
6263.6021	.0017	1.08	40	121069	1	6*p*	114805	2	3*d*′	1596.0894
6284.3870	.0073	0.80	92	123468	1	6*d*	117184	2	5*p*	1590.8105
6287.7156	.0003	3.84	34	121655	2	4*f*′	115367	1	3*d*′	1589.9683
6294.2547	.0003	3.22	28	113426	2	3*d*	107132	1	4*p*′	1588.3165
6320.6730	.0003	2.84	31	118460	1	5*p*′	112139	2	3*d*	1581.6779
6330.1276	.0003	2.39	30	118469	2	5*p*′	112139	2	3*d*	1579.3155
6336.7646	.0003	2.24	29	113468	2	5*s*	107132	1	4*p*′	1577.6613
6353.5597	.0003	2.75	29	113643	1	5*s*	107290	2	4*p*′	1573.4909
6354.1562	.0016	1.04	35	123505	2	5*d*′	117151	1	5*p*	1573.3432
6426.8546	.0003	2.55	28	113717	3	3*d*′	107290	2	4*p*′	1555.5460
6465.4784	.0020	0.97	37	121271	2	6*p*	114805	2	3*d*′	1546.2534
6472.0932	.0003	2.49	32	120189	2	4*f*	113717	3	3*d*	1544.6731
6490.6200	.0003	3.67	32	120207	4	4*f*	113717	3	3*d*	1540.2640
6495.2257	.0020	1.02	41	121470	0	6*p*	114975	1	5*s*′	1539.1718
6511.5511	.0003	3.50	30	113643	1	5*s*	107132	1	4*p*′	1535.3128
6513.1985	.0003	3.67	32	120230	3	4*f*	113717	3	3*d*	1534.9245
6513.5093	.0003	2.03	35	120230	2	4*f*	113717	3	3*d*	1534.8513
6521.6538	.0003	3.79	29	112139	2	3*d*	105617	2	4*p*	1532.9345
6533.3600	.0003	4.32	33	120250	4	4*f*	113717	3	3*d*	1530.1878
6544.9749	.0003	1.98	32	120188	1	4*f*	113643	1	5*s*	1527.4723
6545.3873	.0008	1.49	47	120189	2	4*f*	113643	1	5*s*	1527.3761
6558.0881	.0020	1.26	72	123557	3	5*d*′	116999	2	5*p*	1524.4181
6586.8027	.0003	2.24	35	120230	2	4*f*	113643	1	5*s*	1517.7725
6588.9881	.0003	4.27	30	113643	1	5*s*	107054	0	4*p*	1517.2691
6616.1631	.0020	1.17	60	121257	1	6*p*	114641	2	3*d*′	1511.0371
6641.5687	.0004	1.78	32	118460	1	5*p*′	111818	1	3*d*	1505.2570
6644.2443	.0003	4.82	31	115367	1	3*d*′	108723	0	4*p*′	1504.6505
6651.0239	.0003	2.57	32	118469	2	5*p*′	111818	1	3*d*	1503.1171
6651.3147	.0003	3.12	30	114148	1	3*d*	107496	1	4*p*′	1503.0514
6676.1637	.0003	1.87	30	112139	2	3*d*	105463	3	4*p*	1497.4569
6679.5614	.0003	2.16	34	121655	2	4*f*′	114975	1	5*s*′	1496.6952
6710.4045	.0012	1.45	65	123653	4	6*d*	116943	3	5*p*	1489.8159
6719.7626	.0005	1.58	31	120188	1	4*f*	113468	2	5*s*	1487.7412
6720.1741	.0003	2.38	34	120189	2	4*f*	113468	2	5*s*	1487.6501
6739.6641	.0003	2.10	33	118407	1	5*p*′	111668	0	3*d*	1483.3481
6761.2791	.0003	2.92	33	120230	3	4*f*	113468	2	5*s*	1478.6060
6761.5892	.0003	2.09	34	120230	2	4*f*	113468	2	5*s*	1478.5382
6762.2723	.0003	2.24	33	120188	1	4*f*	113426	2	3*d*	1478.3888
6762.6812	.0008	1.44	41	120189	2	4*f*	113426	2	3*d*	1478.2994
6781.4033	.0005	1.64	38	120250	3	4*f*	113468	2	5*s*	1474.2181
6782.8037	.0003	3.52	30	113020	3	3*d*	106238	2	4*p*	1473.9137
6791.8316	.0003	2.23	32	118460	1	5*p*′	111668	0	3*d*	1471.9546
6803.7904	.0003	2.41	34	120230	3	4*f*	113426	2	3*d*	1469.3674
6804.1004	.0003	3.49	35	120230	2	4*f*	113426	2	3*d*	1469.3004
6807.9810	.0018	1.34	78	123468	1	6*d*	116660	1	5*p*	1468.4629
6823.9129	.0003	4.15	34	120250	3	4*f*	113426	2	3*d*	1465.0345
6831.3425	.0003	4.14	34	121653	4	4*f*′	114822	3	3*d*′	1463.4411
6832.2944	.0003	2.81	33	121654	3	4*f*′	114822	3	3*d*′	1463.2372
6832.6383	.0004	1.91	37	121655	2	4*f*′	114822	3	3*d*′	1463.1645
6848.1066	.0003	2.10	34	121653	3	4*f*′	114805	2	3*d*′	1459.8586
6849.0986	.0003	3.90	34	121654	3	4*f*′	114805	2	3*d*′	1459.6472
6849.4457	.0003	2.67	35	121655	2	4*f*′	114805	2	3*d*′	1459.5732
6858.0314	.0003	2.68	31	114148	1	3*d*	107290	2	4*p*′	1457.7459
6960.4610	.0016	1.05	36	123903	2	8*s*	116943	3	5*p*	1436.2938
7012.2491	.0003	3.92	35	121653	3	4*f*′	114641	2	3*d*′	1425.6862
7013.2417	.0007	1.42	35	121654	3	4*f*′	114641	2	3*d*′	1425.4845
7013.5887	.0003	2.70	35	121655	2	4*f*′	114641	2	3*d*′	1425.4139
7016.0228	.0003	3.86	32	114148	1	3*d*	107132	1	4*p*′	1424.9194
7052.8888	.0003	2.11	34	118871	0	5*p*′	111818	1	3*d*	1417.4712
7093.4598	.0003	4.80	33	114148	1	3*d*	107054	0	4*p*	1409.3640
7109.4333	.0010	1.42	51	121257	1	6*p*	114148	1	3*d*	1406.1974
7144.5754	.0003	2.55	31	114641	2	3*d*′	107496	1	4*p*′	1399.2807
7168.2913	.0008	1.45	40	120189	2	4*f*	113020	3	3*d*	1394.6513
7186.8195	.0003	4.16	36	120207	4	4*f*	113020	3	3*d*	1391.0558
7188.4115	.0003	3.60	32	113426	2	3*d*	106238	2	4*p*	1390.7477
7209.3980	.0003	2.40	35	120230	3	4*f*	113020	3	3*d*	1386.6992
7209.7084	.0003	2.24	38	120230	2	4*f*	113020	3	3*d*	1386.6395
7229.5208	.0003	3.32	36	120250	3	4*f*	113020	3	3*d*	1382.8394
7229.5596	.0003	2.97	36	120250	4	4*f*	113020	3	3*d*	1382.8320
7230.9218	.0003	4.20	33	113468	2	5*s*	106238	2	4*p*	1382.5715
7287.3941	.0003	5.47	36	112750	4	3*d*	105463	3	4*p*	1371.8575
7308.7184	.0003	4.92	33	114805	2	3*d*′	107496	1	4*p*′	1367.8549
7319.5339	.0008	1.69	75	122686	2	5*f*	115367	1	3*d*′	1365.8337 B
7322.4961	.0007	1.58	47	121470	0	6*p*	114148	1	3*d*	1365.2812
7338.7045	.0003	5.07	33	113426	2	3*d*	106087	1	4*p*	1362.2658
7351.2921	.0003	4.23	33	114641	2	3*d*′	107290	2	4*p*′	1359.9332
7365.2190	.0003	3.89	34	114862	0	5*s*′	107496	1	4*p*′	1357.3617
7381.2145	.0003	3.74	33	113468	2	5*s*	106087	1	4*p*	1354.4202
7403.0851	.0003	5.34	35	113020	3	3*d*	105617	2	4*p*	1350.4189
7405.7081	.0003	4.18	34	113643	1	5*s*	106238	2	4*p*	1349.9406
7448.8049	.0014	1.18	40	121165	3	6*p*	113717	3	3*d*	1342.1302
7456.9807	.0003	4.39	37	120207	5	4*f*	112750	4	3*d*	1340.6587
7475.2491	.0012	1.35	51	121192	2	6*p*	113717	3	3*d*	1337.3823
7478.6019	.0003	3.94	34	114975	1	5*s*′	107496	1	4*p*′	1336.7827
7479.0030	.0003	5.23	35	113717	3	3*d*	106238	2	4*p*	1336.7110
7479.5986	.0003	2.14	38	120230	3	4*f*	112750	4	3*d*	1336.6046
7499.7604	.0003	3.41	37	120250	4	4*f*	112750	4	3*d*	1333.0114
7506.8488	.0003	2.11	38	121655	2	4*f*′	114148	1	3*d*	1331.7527
7509.2835	.0003	5.02	34	114641	2	3*d*′	107132	1	4*p*′	1331.3209
7515.4349	.0003	3.45	33	114805	2	3*d*′	107290	2	4*p*′	1330.2312
7532.2390	.0003	5.18	35	114822	3	3*d*′	107290	2	4*p*′	1327.2635
7548.5433	.0004	1.81	41	121192	2	6*p*	113643	1	5*s*	1324.3967
7554.0597	.0004	1.96	45	121271	2	6*p*	113717	3	3*d*	1323.4295
7556.0010	.0003	4.42	34	113643	1	5*s*	106087	1	4*p*	1323.0895
7557.5951	.0003	4.63	34	113020	3	3*d*	105463	3	4*p*	1322.8104
7565.6667	.0003	4.59	33	111668	0	3*d*	104102	1	4*p*	1321.3992
7600.2631	.0008	1.55	49	121069	1	6*p*	113468	2	5*s*	1315.3842
7613.8980	.0005	1.70	39	121257	1	6*p*	113643	1	5*s*	1313.0286
7626.2064	.0071	0.46	41	122601	1	6*p*′	114975	1	5*s*′	1310.9094
7627.3559	.0010	1.36	44	121271	2	6*p*	113643	1	5*s*	1310.7118
7660.0431	.0006	1.63	44	122635	2	6*p*′	114975	1	5*s*′	1305.1187
7673.4263	.0003	3.23	34	114805	2	3*d*′	107132	1	4*p*′	1302.8424
7685.3185	.0003	4.60	35	114975	1	5*s*′	107290	2	4*p*′	1300.8264
7696.8884	.0004	2.07	43	121165	3	6*p*	113468	2	5*s*	1298.8710
7715.9287	.0003	4.94	34	111818	1	3*d*	104102	1	4*p*	1295.6659
7729.9269	.0003	4.09	35	114862	0	5*s*′	107132	1	4*p*′	1293.3195
7748.0090	.0131	0.16	38	122610	1	6*p*′	114862	0	5*s*′	1290.3012
7765.8417	.0010	1.45	51	121192	2	6*p*	113426	2	3*d*	1287.3383
7802.1377	.0004	1.81	41	121271	2	6*p*	113468	2	5*s*	1281.3495
7808.6929	.0003	4.72	35	113426	2	3*d*	105617	2	4*p*	1280.2738
7813.1258	.0005	1.77	48	122635	2	6*p*′	114822	3	3*d*′	1279.5474
7815.5226	.0012	1.42	62	122791	0	6*p*′	114975	1	5*s*′	1279.1550
7826.9723	.0005	1.78	46	121470	0	6*p*	113643	1	5*s*	1277.2838
7831.1933	.0006	1.68	50	121257	1	6*p*	113426	2	3*d*	1276.5954
7843.3100	.0003	3.86	36	114975	1	5*s*′	107132	1	4*p*′	1274.6232
7851.2029	.0003	4.04	35	113468	2	5*s*	105617	2	4*p*	1273.3418
7870.4485	.0003	4.64	36	115367	1	3*d*′	107496	1	4*p*′	1270.2281
7873.7454	.0047	0.74	51							1269.6962
7881.2694	.0037	0.88	56	122686	2	5*f*	114805	2	3*d*′	1268.4841
7895.9030	.0065	0.91	105	122718	4	5*f*	114822	3	3*d*′	1266.1332 B
7902.7445	.0047	0.74	51	122708	3	5*f*	114805	2	3*d*′	1265.0371
7910.1795	.0003	2.89	36	114148	1	3*d*	106238	2	4*p*	1263.8480
7920.7469	.0003	3.15	36	114975	1	5*s*′	107054	0	4*p*	1262.1619
7936.7267	.0003	2.65	39	121653	4	4*f*′	113717	3	3*d*	1259.6206
7937.6794	.0008	1.31	31	121654	3	4*f*′	113717	3	3*d*	1259.4695
7963.2029	.0003	3.11	35	113426	2	3*d*	105463	3	4*p*	1255.4326
7968.6520	.0011	1.27	38	122610	1	6*p*′	114641	2	3*d*′	1254.5741
8005.7130	.0003	4.88	38	113468	2	5*s*	105463	3	4*p*	1248.7663
8025.9896	.0003	4.66	37	113643	1	5*s*	105617	2	4*p*	1245.6114
8036.8250	.0003	4.85	36	112139	2	3*d*	104102	1	4*p*	1243.9321
8049.3114	.0003	2.25	38	120188	1	4*f*	112139	2	3*d*	1242.0024
8049.7243	.0003	3.23	39	120189	2	4*f*	112139	2	3*d*	1241.9387
8060.4725	.0003	4.78	37	114148	1	3*d*	106087	1	4*p*	1240.2827
8076.8285	.0027	1.10	68	122718	3	5*f*	114641	2	3*d*′	1237.7710
8090.8295	.0003	3.78	39	120230	3	4*f*	112139	2	3*d*	1235.6291
8091.1398	.0003	2.66	40	120230	2	4*f*	112139	2	3*d*	1235.5817
8099.2843	.0003	4.22	36	113717	3	3*d*	105617	2	4*p*	1234.3392
8145.0037	.0022	1.08	52	121165	3	6*p*	113020	3	3*d*	1227.4106
8171.4491	.0004	1.98	46	121192	2	6*p*	113020	3	3*d*	1223.4383
8185.7581	.0012	1.37	56	121654	3	4*f*′	113468	2	5*s*	1221.2997
8227.2780	.0003	3.02	40	121653	3	4*f*′	113426	2	3*d*	1215.1363
8228.2703	.0006	1.48	33	121654	3	4*f*′	113426	2	3*d*	1214.9898
8228.6167	.0005	1.72	40	121655	2	4*f*′	113426	2	3*d*	1214.9386
8235.1565	.0003	4.34	38	115367	1	3*d*′	107132	1	4*p*′	1213.9738
8253.7944	.0003	4.50	37	113717	3	3*d*	105463	3	4*p*	1211.2325
8312.5935	.0003	3.38	38	115367	1	3*d*′	107054	0	4*p*	1202.6648
8370.2083	.0003	3.26	40	120188	1	4*f*	111818	1	3*d*	1194.3865
8370.6209	.0003	3.51	41	120189	2	4*f*	111818	1	3*d*	1194.3276
8403.4405	.0003	2.80	37	114641	2	3*d*′	106238	2	4*p*	1189.6632
8412.0363	.0003	3.00	42	120230	2	4*f*	111818	1	3*d*	1188.4475
8415.2098	.0004	2.10	46	121165	3	6*p*	112750	4	3*d*	1187.9993
8520.4703	.0003	3.32	40	120188	1	4*f*	111668	0	3*d*	1173.3229
8530.4611	.0003	3.61	38	114148	1	3*d*	105617	2	4*p*	1171.9488
8538.3141	.0034	0.95	60	122686	1	5*f*	114148	1	3*d*	1170.8709
8538.6632	.0013	1.62	103	122686	2	5*f*	114148	1	3*d*	1170.8230
8553.7333	.0003	2.65	38	114641	2	3*d*′	106087	1	4*p*	1168.7602
8560.3877	.0005	2.38	158	122708	2	5*f*	114148	1	3*d*	1167.8517
8567.5832	.0003	4.33	38	114805	2	3*d*′	106238	2	4*p*	1166.8709
8584.3886	.0013	1.18	37	114822	3	3*d*′	106238	2	4*p*	1164.5865
8632.9262	.0003	2.59	42	121653	4	4*f*′	113020	3	3*d*	1158.0387
8633.8790	.0011	1.32	44	121654	3	4*f*′	113020	3	3*d*	1157.9109
8702.2714	.0003	4.13	39	104102	1	4*p*	95400	1	4*s*′	1148.8107
8717.8762	.0003	3.34	38	114805	2	3*d*′	106087	1	4*p*	1146.7544
8737.4668	.0003	3.70	39	114975	1	5*s*′	106238	2	4*p*	1144.1832
8770.5680	.0093	0.79	115	124137	2	5*f*′	115367	1	3*d*′	1139.8649
8774.3768	.0004	3.31	39	114862	0	5*s*′	106087	1	4*p*	1139.3701
8887.7599	.0004	2.03	40	114975	1	5*s*′	106087	1	4*p*	1124.8349
8929.8069	.0025	0.90	40	121069	1	6*p*	112139	2	3*d*	1119.5384
8979.1216	.0014	1.61	112	122696	4	5*f*	113717	3	3*d*	1113.3898
8991.3269	.0228	0.42	120	122708	3	5*f*	113717	3	3*d*	1111.8784
9001.2763	.0008	2.10	166	122718	3	5*f*	113717	3	3*d*	1110.6494
9023.7218	.0004	3.63	40	114641	2	3*d*′	105617	2	4*p*	1107.8868
9026.4358	.0029	0.89	45	121165	3	6*p*	112139	2	3*d*	1107.5536
9118.2337	.0024	0.97	45	121257	1	6*p*	112139	2	3*d*	1096.4033
9129.3133	.0004	3.24	41	115367	1	3*d*′	106238	2	4*p*	1095.0727
9131.6914	.0005	1.81	49	121271	2	6*p*	112139	2	3*d*	1094.7875
9175.2882	.0004	2.17	45							1089.5855
9178.2318	.0004	2.55	40	114641	2	3*d*′	105463	3	4*p*	1089.2361
9187.8647	.0004	3.39	40	114805	2	3*d*′	105617	2	4*p*	1088.0941
9204.6688	.0004	2.35	41	114822	3	3*d*′	105617	2	4*p*	1086.1077
9250.7077	.0011	1.37	48	121069	1	6*p*	111818	1	3*d*	1080.7023
9279.6063	.0004	2.63	42	115367	1	3*d*′	106087	1	4*p*	1077.3368
9282.1503	.0037	1.33	158	122708	2	5*f*	113426	2	3*d*	1077.0415
9313.7480	.0007	2.03	130	124136	4	5*f*′	114822	3	3*d*′	1073.3875
9315.2458	.0076	0.85	107	124137	3	5*f*′	114822	3	3*d*′	1073.2149 ?
9323.8646	.0005	1.74	41	113426	2	3*d*	104102	1	4*p*	1072.2229
9332.0600	.0011	1.80	124	124137	3	5*f*′	114805	2	3*d*′	1071.2813
9342.3745	.0004	2.87	41	114805	2	3*d*′	105463	3	4*p*	1070.0985
9357.7483	.0004	2.55	42	114975	1	5*s*′	105617	2	4*p*	1068.3404
9359.1787	.0004	3.80	41	114822	3	3*d*′	105463	3	4*p*	1068.1771
9366.3742	.0004	4.52	43	113468	2	5*s*	104102	1	4*p*	1067.3565
9400.9699	.0020	1.18	60	121069	1	6*p*	111668	0	3*d*	1063.4286
9439.1217	.0012	1.33	47	121257	1	6*p*	111818	1	3*d*	1059.1304
9452.5840	.0022	1.15	61	121271	2	6*p*	111818	1	3*d*	1057.6220
9494.6660	.0005	2.14	77	124136	3	5*f*′	114641	2	3*d*′	1052.9344
9515.3098	.0004	2.95	45	121654	3	4*f*′	112139	2	3*d*	1050.6500
9515.6569	.0005	1.84	48	121655	2	4*f*′	112139	2	3*d*	1050.6117
9541.1608	.0004	3.95	43	113643	1	5*s*	104102	1	4*p*	1047.8034
9548.4347	.0004	4.73	42	104102	1	4*p*	94554	0	4*s*′	1047.0051
9675.3296	.0005	2.39	113	122696	4	5*f*	113020	3	3*d*	1033.2743
9697.4736	.0021	1.57	151	122718	3	5*f*	113020	3	3*d*	1030.9155
9749.5947	.0004	2.06	45	115367	1	3*d*′	105617	2	4*p*	1025.4026
9836.5531	.0004	2.27	48	121655	2	4*f*′	111818	1	3*d*	1016.3377
9928.7757	.0004	2.21	45	118651	1	4*d*	108723	0	4*p*′	1006.8975
9945.4830	.0004	2.57	114	122696	5	5*f*	112750	4	3*d*	1005.2060
9967.6693	.0033	1.45	182	122718	3	5*f*	112750	4	3*d*	1002.9686
10200.3194	.0055	0.70	55	123221	2	7*p*	113020	3	3*d*	980.0927
10217.4425	.0004	4.55	49	105617	2	4*p*	95400	1	4*s*′	978.4502
10341.7253	.0072	0.90	114	124058	3	6*f*	113717	3	3*d*	966.6915 B
10351.5020	.0004	4.80	58	104102	1	4*p*	93751	1	4*s*	965.7785
10419.1483	.0062	0.75	69	124136	4	5*f*′	113717	3	3*d*	959.5081
10455.6379	.0034	0.87	50	123206	3	7*p*	112750	4	3*d*	956.1595
10547.4896	.0012	1.58	84	122686	2	5*f*	112139	2	3*d*	947.8328
10569.2113	.0045	0.95	79	122708	2	5*f*	112139	2	3*d*	945.8849
10632.2932	.0082	0.90	130	124058	3	6*f*	113426	2	3*d*	940.2729 B
10660.7525	.0258	0.68	247	125483	4	6*f*′	114822	3	3*d*′	937.7628
10678.0032	.0110	0.62	92	125483	3	6*f*′	114805	2	3*d*′	936.2478
10687.4309	.0004	4.14	48	106087	1	4*p*	95400	1	4*s*′	935.4219
10703.0310	.0031	0.90	48	114805	2	3*d*′	104102	1	4*p*	934.0579
10709.7104	.0031	1.11	80	124136	3	5*f*′	113426	2	3*d*	933.4763
10759.5356	.0004	3.02	47	114862	0	5*s*′	104102	1	4*p*	929.1530
10837.7237	.0004	4.62	63	106238	2	4*p*	95400	1	4*s*′	922.4498
10841.6683	.0175	0.68	167	125483	3	6*f*′	114641	2	3*d*′	922.1142 B
10868.0457	.0010	1.61	71	122686	1	5*f*	111818	1	3*d*	919.8770
10868.3873	.0007	1.86	71	122686	2	5*f*	111818	1	3*d*	919.8479
10872.9204	.0004	3.27	47	114975	1	5*s*′	104102	1	4*p*	919.4637
10890.1131	.0023	1.25	81	122708	2	5*f*	111818	1	3*d*	918.0127
10958.3386	.0007	5.89	71	104102	1	4*p*	93144	2	4*s*	912.2990
11015.7819	.0005	2.13	49	118512	0	4*d*	107496	1	4*p*′	907.5393
11018.3070	.0009	1.64	70	122686	1	5*f*	111668	0	3*d*	907.3319
11037.5531	.0027	1.03	57	119760	1	6*s*	108723	0	4*p*′	905.7492
11154.9795	.0009	1.52	48	118651	1	4*d*	107496	1	4*p*′	896.2145
11264.7696	.0030	0.86	43	115367	1	3*d*′	104102	1	4*p*	887.4797
11296.4703	.0036	1.34	157	124047	5	6*f*	112750	4	3*d*	884.9917 ?
11361.6963	.0005	1.97	49	118651	1	4*d*	107290	2	4*p*′	879.9086
11380.4908	.0017	1.23	54	118512	0	4*d*	107132	1	4*p*′	878.4554
11410.1956	.0005	2.39	49	118907	2	4*d*	107496	1	4*p*′	876.1685
11519.6873	.0006	1.74	49	118651	1	4*d*	107132	1	4*p*′	867.8407
11533.5942	.0005	4.18	51	106087	1	4*p*	94554	0	4*s*′	866.7943
11597.1244	.0005	2.19	50	118651	1	4*d*	107054	0	4*p*	862.0459
11616.9120	.0005	2.37	50	118907	2	4*d*	107290	2	4*p*′	860.5775
11654.4522	.0032	0.86	46	107054	0	4*p*	95400	1	4*s*′	857.8055
11731.8805	.0005	4.28	73	107132	1	4*p*′	95400	1	4*s*′	852.1441
11774.9031	.0011	1.38	46	118907	2	4*d*	107132	1	4*p*′	849.0306
11840.2156	.0157	0.55	111	124861	4	7*f*	113020	3	3*d*	844.3472 ?
11866.6727	.0005	4.27	112	105617	2	4*p*	93751	1	4*s*	842.4647 R
11889.8716	.0005	4.25	105	107290	2	4*p*′	95400	1	4*s*′	840.8209 R
11912.4488	.0055	0.93	94	124051	3	6*f*	112139	2	3*d*	839.2273 ?
11912.7258	.0130	0.22	43	124052	2	6*f*	112139	2	3*d*	839.2078 B
11923.1757	.0007	1.66	51	119213	3	4*d*	107290	2	4*p*′	838.4723
11998.2807	.0071	0.77	83	124137	3	5*f*′	112139	2	3*d*	833.2237
12096.5884	.0005	4.16	76	107496	1	4*p*′	95400	1	4*s*′	826.4521
12186.6678	.0028	1.00	55	119683	2	6*s*	107496	1	4*p*′	820.3433
12313.1259	.0010	1.50	54	119445	2	4*d*	107132	1	4*p*′	811.9182
12318.9991	.0005	4.22	141	105463	3	4*p*	93144	2	4*s*	811.5311 R
12336.6612	.0005	4.22	100	106087	1	4*p*	93751	1	4*s*	810.3692 R
12351.3487	.0027	0.97	50	119848	1	4*d*	107496	1	4*p*′	809.4056
12393.3829	.0020	1.12	51	119683	2	6*s*	107290	2	4*p*′	806.6603
12413.8443	.0005	2.08	53	118651	1	4*d*	106238	2	4*p*	805.3307
12424.9399	.0008	1.60	53	118512	0	4*d*	106087	1	4*p*	804.6115
12438.6957	.0017	1.28	62	121161	1	6*s*′	108723	0	4*p*′	803.7217
12473.5104	.0005	4.32	87	105617	2	4*p*	93144	2	4*s*	801.4785 R
12486.9545	.0005	4.24	66	106238	2	4*p*	93751	1	4*s*	800.6155
12578.0436	.0005	4.25	83	107132	1	4*p*′	94554	0	4*s*′	794.8175 R
12628.4665	.0023	1.08	55	119760	1	6*s*	107132	1	4*p*′	791.6440
12669.0604	.0005	2.19	53	118907	2	4*d*	106238	2	4*p*	789.1074
12705.9011	.0012	1.42	56	119760	1	6*s*	107054	0	4*p*	786.8194
12716.0561	.0032	0.95	57	119848	1	4*d*	107132	1	4*p*′	786.1910
12793.4975	.0047	0.86	68	119848	1	4*d*	107054	0	4*p*	781.4326
12819.3559	.0016	1.22	52	118907	2	4*d*	106087	1	4*p*	779.8556
12942.7518	.0005	4.16	73	107496	1	4*p*′	94554	0	4*s*′	772.4206
12943.4991	.0005	4.21	63	106087	1	4*p*	93144	2	4*s*	772.3760
12975.3203	.0023	1.00	45	119213	3	4*d*	106238	2	4*p*	770.4818
13034.1248	.0009	1.54	55	118651	1	4*d*	105617	2	4*p*	767.0057
13093.7911	.0005	4.19	123	106238	2	4*p*	93144	2	4*s*	763.5105 R
13104.4750	.0010	1.52	59	120601	2	4*d*′	107496	1	4*p*′	762.8880
13122.6038	.0008	1.77	64	120619	2	4*d*′	107496	1	4*p*′	761.8335 B
13210.2160	.0058	0.82	76	121933	1	5*d*	108723	0	4*p*′	756.7815
13303.6742	.0005	4.23	80	107054	0	4*p*	93751	1	4*s*	751.4651
13311.1897	.0009	1.61	54	120601	2	4*d*′	107290	2	4*p*′	751.0408
13322.7911	.0005	4.35	75	108723	0	4*p*′	95400	1	4*s*′	750.3868
13328.4986	.0017	1.26	57	119566	3	4*d*	106238	2	4*p*	750.0655
13357.5779	.0007	1.81	56	119445	2	4*d*	106087	1	4*p*	748.4326
13381.1108	.0006	2.05	54	107132	1	4*p*′	93751	1	4*s*	747.1163
13443.8523	.0008	1.69	54	118907	2	4*d*	105463	3	4*p*	743.6297
13445.5300	.0006	2.15	56	119683	2	6*s*	106238	2	4*p*	743.5369
13463.7726	.0008	1.64	56	120753	3	4*d*′	107290	2	4*p*′	742.5294
13469.1842	.0040	0.87	58	120601	2	4*d*′	107132	1	4*p*′	742.2311
13487.3079	.0008	1.68	55	120619	2	4*d*′	107132	1	4*p*′	741.2337
13522.6205	.0007	1.87	56	119760	1	6*s*	106238	2	4*p*	739.2980
13539.1027	.0005	4.26	82	107290	2	4*p*′	93751	1	4*s*	738.3980
13560.8872	.0005	2.81	56	119024	4	4*d*	105463	3	4*p*	737.2118
13595.6041	.0006	2.36	57	119213	3	4*d*	105617	2	4*p*	735.3293
13595.8213	.0016	1.25	54	119683	2	6*s*	106087	1	4*p*	735.3176
13600.1892	.0015	1.27	52	121097	0	6*s*′	107496	1	4*p*′	735.0814
13664.8971	.0007	1.84	57	121161	1	6*s*′	107496	1	4*p*′	731.6006
13672.9133	.0006	2.07	57	119760	1	6*s*	106087	1	4*p*	731.1716
13722.2228	.0029	1.03	60	121012	1	4*d*′	107290	2	4*p*′	728.5442
13745.8191	.0005	3.80	58	107496	1	4*p*′	93751	1	4*s*	727.2936
13750.1138	.0011	1.46	53	119213	3	4*d*	105463	3	4*p*	727.0664
13760.5079	.0010	1.55	62	119848	1	4*d*	106087	1	4*p*	726.5172
13827.5643	.0033	0.91	53	119445	2	4*d*	105617	2	4*p*	722.9943
13871.6141	.0006	2.27	57	121161	1	6*s*′	107290	2	4*p*′	720.6981
13957.6474	.0038	0.98	71	121012	1	4*d*′	107054	0	4*p*	716.2561
13964.8985	.0009	1.65	61	121097	0	6*s*′	107132	1	4*p*′	715.8838
13987.9487	.0006	3.50	56	107132	1	4*p*′	93144	2	4*s*	714.7041
14029.6054	.0008	1.77	59	121161	1	6*s*′	107132	1	4*p*′	712.5820
14065.8104	.0007	1.85	57	119683	2	6*s*	105617	2	4*p*	710.7478
14107.0439	.0022	1.10	53	121161	1	6*s*′	107054	0	4*p*	708.6704
14142.9014	.0006	2.42	58	119760	1	6*s*	105617	2	4*p*	706.8736
14145.9401	.0006	4.23	68	107290	2	4*p*′	93144	2	4*s*	706.7218
14220.3212	.0006	2.62	59	119683	2	6*s*	105463	3	4*p*	703.0252
14297.6829	.0026	1.10	64	121794	0	5*d*	107496	1	4*p*′	699.2212
14352.6567	.0006	4.17	70	107496	1	4*p*′	93144	2	4*s*	696.5430
14363.3400	.0011	1.48	59	120601	2	4*d*′	106238	2	4*p*	696.0250
14381.4656	.0012	1.44	59	120619	2	4*d*′	106238	2	4*p*	695.1477
14410.0995	.0006	2.34	59	118512	0	4*d*	104102	1	4*p*	693.7664
14513.6311	.0010	1.56	59	120601	2	4*d*′	106087	1	4*p*	688.8175
14515.9204	.0017	1.22	53	120753	3	4*d*′	106238	2	4*p*	688.7088
14531.7581	.0014	1.37	59	120619	2	4*d*′	106087	1	4*p*	687.9582
14549.2966	.0006	2.74	59	118651	1	4*d*	104102	1	4*p*	687.1289
14643.1510	.0030	1.06	68	121933	1	5*d*	107290	2	4*p*′	682.7248
14774.3674	.0011	1.52	62	121012	1	4*d*′	106238	2	4*p*	676.6612
14797.2184	.0019	1.27	66	122087	2	5*d*	107290	2	4*p*′	675.6165
14801.1458	.0057	0.75	64	121933	1	5*d*	107132	1	4*p*′	675.4370
14804.5129	.0006	2.72	60	118907	2	4*d*	104102	1	4*p*	675.2834
14878.5769	.0030	1.07	70	121933	1	5*d*	107054	0	4*p*	671.9218
14923.7635	.0022	1.16	61	121161	1	6*s*′	106238	2	4*p*	669.8874
14972.0217	.0006	2.62	59	108723	0	4*p*′	93751	1	4*s*	667.7281
15001.7457	.0017	1.28	59	120619	2	4*d*′	105617	2	4*p*	666.4051
15009.3499	.0020	1.15	55	121097	0	6*s*′	106087	1	4*p*	666.0675
15136.2016	.0010	1.60	61	120753	3	4*d*′	105617	2	4*p*	660.4854
15290.7116	.0016	1.33	61	120753	3	4*d*′	105463	3	4*p*	653.8112
15394.6522	.0056	0.81	72	121012	1	4*d*′	105617	2	4*p*	649.3968
15459.9161	.0085	0.78	102	122514	1	5*d*	107054	0	4*p*	646.6554
15544.0411	.0039	0.88	59	121161	1	6*s*′	105617	2	4*p*	643.1556
15580.9823	.0006	2.35	64	119683	2	6*s*	104102	1	4*p*	641.6307
15658.0726	.0008	1.75	63	119760	1	6*s*	104102	1	4*p*	638.4717
15695.2938	.0020	1.27	69	121933	1	5*d*	106238	2	4*p*	636.9576
15706.8445	.0044	0.94	75	121794	0	5*d*	106087	1	4*p*	636.4892
15849.3651	.0012	1.50	67	122087	2	5*d*	106238	2	4*p*	630.7657
15876.5080	.0020	1.33	83	123373	2	5*d*′	107496	1	4*p*′	629.6866
16083.2263	.0029	1.08	68	123373	2	5*d*′	107290	2	4*p*′	621.5939
16092.1163	.0017	1.36	71	122330	3	5*d*	106238	2	4*p*	621.2505
16194.8413	.0015	1.40	66	122282	2	5*d*	106087	1	4*p*	617.3098
16202.5128	.0020	1.26	69	122440	2	7*s*	106238	2	4*p*	617.0175
16215.7974	.0077	0.70	77	123505	2	5*d*′	107290	2	4*p*′	616.5120
16241.8345	.0046	0.93	77	122479	1	7*s*	106238	2	4*p*	615.5237
16267.7212	.0017	1.32	66	123557	3	5*d*′	107290	2	4*p*′	614.5442
16373.7785	.0016	1.36	68	123505	2	5*d*′	107132	1	4*p*′	610.5636
16376.5857	.0057	0.83	76	123873	0	7*s*′	107496	1	4*p*′	610.4589
16392.1213	.0025	1.20	77	122479	1	7*s*	106087	1	4*p*	609.8804
16498.7909	.0009	1.74	67	120601	2	4*d*′	104102	1	4*p*	605.9373
16516.9178	.0015	1.41	69	120619	2	4*d*′	104102	1	4*p*	605.2723
16542.8781	.0008	1.93	70	122160	3	5*d*	105617	2	4*p*	604.3224
16573.3095	.0007	2.33	69	122036	4	5*d*	105463	3	4*p*	603.2128
16592.5002	.0035	0.97	65	123882	1	7*s*′	107290	2	4*p*′	602.5151
16624.1544	.0044	0.87	65	122087	2	5*d*	105463	3	4*p*	601.3678
16697.3880	.0026	1.10	62	122160	3	5*d*	105463	3	4*p*	598.7303
16822.7950	.0039	0.93	66	122440	2	7*s*	105617	2	4*p*	594.2669
16862.1077	.0015	1.43	75	122479	1	7*s*	105617	2	4*p*	592.8814
16909.8197	.0008	1.97	70	121012	1	4*d*′	104102	1	4*p*	591.2086
16977.3094	.0012	1.70	105	122440	2	7*s*	105463	3	4*p*	588.8580
16994.5069	.0015	1.39	66	121097	0	6*s*′	104102	1	4*p*	588.2624
17059.2146	.0014	1.44	68	121161	1	6*s*′	104102	1	4*p*	586.0311
17135.3729	.0036	0.99	69	123373	2	5*d*′	106238	2	4*p*	583.4264
17319.8714	.0076	0.89	117	123557	3	5*d*′	106238	2	4*p*	577.2118
17418.2292	.0019	1.26	66	123505	2	5*d*′	106087	1	4*p*	573.9520
17692.0003	.0011	1.65	74	121794	0	5*d*	104102	1	4*p*	565.0704
17830.7477	.0008	1.95	72	121933	1	5*d*	104102	1	4*p*	560.6734
17940.1488	.0018	1.36	78	123557	3	5*d*′	105617	2	4*p*	557.2543
17984.8161	.0009	1.86	73	122087	2	5*d*	104102	1	4*p*	555.8703
18156.5910	.0043	1.02	88	123774	3	6*d*	105617	2	4*p*	550.6113 B
18190.4151	.0012	1.66	82	123653	4	6*d*	105463	3	4*p*	549.5875
18337.9668	.0020	1.33	79	122440	2	7*s*	104102	1	4*p*	545.1655
19147.0962	.0060	1.03	128	124610	4	7*d*	105463	3	4*p*	522.1271
19270.8283	.0016	1.51	87	123373	2	5*d*′	104102	1	4*p*	518.7746
19365.8730	.0018	1.41	81	123468	1	6*d*	104102	1	4*p*	516.2284
19406.8306	.0052	0.95	91	123509	0	6*d*	104102	1	4*p*	515.1390
19757.0357	.0198	0.74	217	125220	4	8*d*	105463	3	4*p*	506.0078
21260.1678	.0012	1.67	85	116660	1	5*p*	95400	1	4*s*′	470.2316
21599.5004	.0021	1.35	85	116999	2	5*p*	95400	1	4*s*′	462.8441
21751.5042	.0016	1.54	90	117151	1	5*p*	95400	1	4*s*′	459.6096
22106.3349	.0015	1.54	87	116660	1	5*p*	94554	0	4*s*′	452.2322
22163.1303	.0009	2.17	89	117563	0	5*p*	95400	1	4*s*′	451.0733
22909.3977	.0070	0.82	92	116660	1	5*p*	93751	1	4*s*	436.3793
23007.6035	.0012	1.77	93	118407	1	5*p*′	95400	1	4*s*′	434.5168
23059.7718	.0012	1.81	92	118460	1	5*p*′	95400	1	4*s*′	433.5338
23069.2269	.0010	2.25	94	118469	2	5*p*′	95400	1	4*s*′	433.3561
23248.7308	.0010	2.29	94	116999	2	5*p*	93751	1	4*s*	430.0101
23400.7318	.0010	2.38	93	117151	1	5*p*	93751	1	4*s*	427.2169
23432.9967	.0010	2.13	93	117184	2	5*p*	93751	1	4*s*	426.6286
23471.0911	.0010	2.45	95	118871	0	5*p*′	95400	1	4*s*′	425.9362
23516.2369	.0015	1.59	94	116660	1	5*p*	93144	2	4*s*	425.1185
23798.9979	.0010	2.76	96	116943	3	5*p*	93144	2	4*s*	420.0674
23812.3612	.0010	2.50	95	117563	0	5*p*	93751	1	4*s*	419.8317
23853.7677	.0011	1.98	96	118407	1	5*p*′	94554	0	4*s*′	419.1029
23855.5677	.0010	2.13	95	116999	2	5*p*	93144	2	4*s*	419.0713
23905.9348	.0011	1.97	94	118460	1	5*p*′	94554	0	4*s*′	418.1883
24007.5691	.0011	1.89	95	117151	1	5*p*	93144	2	4*s*	416.4179
24039.8337	.0010	2.76	97	117184	2	5*p*	93144	2	4*s*	415.8590
24656.8287	.0103	0.77	121	118407	1	5*p*′	93751	1	4*s*	405.4527
24709.0026	.0065	0.89	100	118460	1	5*p*′	93751	1	4*s*	404.5965
24718.4566	.0011	1.97	99	118469	2	5*p*′	93751	1	4*s*	404.4418
25120.3269	.0085	0.76	71	118871	0	5*p*′	93751	1	4*s*	397.9715
25315.8403	.0013	1.79	99	118460	1	5*p*′	93144	2	4*s*	394.8979
25325.2989	.0034	1.17	96	118469	2	5*p*′	93144	2	4*s*	394.7504
25668.9124	.0063	1.29	86	121069	1	6*p*	95400	1	4*s*′	389.4560
26070.4161	.0060	1.99	84	121470	0	6*p*	95400	1	4*s*′	383.4678
26515.0764	.0063	1.41	86	121069	1	6*p*	94554	0	4*s*′	377.0368
27085.9965	.0066	1.35	86	120230	3	4*f*	93144	2	4*s*	369.0894
27318.1447	.0068	1.25	89	121069	1	6*p*	93751	1	4*s*	365.9529
27390.7207	.0064	1.70	86	122791	0	6*p*′	95400	1	4*s*′	364.9832
27506.5746	.0165	0.52	109	121257	1	6*p*	93751	1	4*s*	363.4459
27520.0268	.0064	1.56	83	121271	2	6*p*	93751	1	4*s*	363.2682
27719.6402	.0091	0.72	95	121470	0	6*p*	93751	1	4*s*	360.6522
27985.2236	.0068	1.33	95	123385	0	7*p*	95400	1	4*s*′	357.2295
28021.6135	.0064	1.96	86	121165	3	6*p*	93144	2	4*s*	356.7655
28126.8637	.0062	0.95	109	121271	2	6*p*	93144	2	4*s*	355.4305
29634.4517	.0075	1.15	87	123385	0	7*p*	93751	1	4*s*	337.3482
30117.7873	.0077	1.12	95	123262	2	7*p*	93144	2	4*s*	331.9342

a The notation *nl* (e.g., 6*p*) denotes levels of the 3*p*^5^*nl* configuration with 3*p*^5^(^2^P_3/2_) core. The notation *nl*′ (e.g., 6*p*′) denotes levels of the 3*p*^5^*nl* configuration with 3*p*^5^(^2^P_1/2_) core.
